# Regulation of antiviral and antitumor immunity by the *BRCA1* pseudogene in human cancers

**DOI:** 10.1073/pnas.2528911123

**Published:** 2026-05-04

**Authors:** Yoo Jane Han, Jing Zhang, Maryam Shariff, Sulin Wu, Galina Khramtsova, Long Chi Nguyen, Daniel S. Peiffer, Nansheng Li, Anna Lewicka, Matthew Moore, Joseph A. Piccirilli, Olufunmilayo I. Olopade

**Affiliations:** ^a^ Section of Hematology/Oncology, Department of Medicine, University of Chicago, Chicago, IL 60637; ^b^ Ben May Department for Cancer Research, University of Chicago, Chicago, IL 60637; ^c^ Department of Biochemistry and Molecular Biology, University of Chicago, Chicago, IL 60637; ^d^ Department of Chemistry, University of Chicago, Chicago, IL 60637; ^e^ Department of Human Genetics, University of Chicago, Chicago, IL 60637

**Keywords:** *BRCA1P1*, pseudogene, circular RNA, antiviral immune response, antitumor immunity

## Abstract

Pseudogenes have been regarded as nonfunctional byproducts of evolutionary processes. However, emerging evidence indicates that pseudogenes perform diverse biological roles in human physiology and pathology. We identified the *BRCA1* pseudogene (*BRCA1P1*), a fusion pseudogene derived from the *BRCA1* tumor suppressor and *RPLP1* ribosomal protein genes, as an immunoregulatory RNA in breast cancer. In this study, we show that *BRCA1P1* expression varies across multiple cancer cell types, with no significant association with *BRCA1* or *BRCA2* somatic mutations in breast and ovarian tumors. Interestingly, *BRCA1P1* inhibition elicits antitumor effects in multiple cancer cell types and preclinical tumor models through an antiviral defense mechanism. Loss of *BRCA1P1* induces antiviral gene expression, promotes apoptosis, and increases sensitivity to chemotherapy in various cancer cells, without inducing apoptosis in nonmalignant cells. This antiviral response also enhances macrophage-mediated phagocytosis of *BRCA1P1*-deficient cancer cells. Mechanistically, the majority of *BRCA1P1* transcripts are circular RNAs and regulate NF-κB-driven antiviral gene expression. Furthermore, intratumoral expression of *BRCA1P1* is elevated in tumor cells, compared to normal breast tissue, and its depletion significantly inhibits the growth of both primary and metastatic breast tumor organoids. Finally, in a humanized mouse model of breast cancer, *BRCA1P1* loss stimulates antiviral gene expression and increases T cell infiltration into tumors. These findings support a critical role for *BRCA1P1* in regulating innate immune defense and antitumor responses across cancer types, suggesting that targeting pseudogene-derived RNAs may offer innovative therapeutic strategies to enhance antitumor immunity in breast and other cancers.

## Significance

This study uncovers a mechanism by which a host-derived pseudogene RNA regulates innate immunity and highlights its clinicopathological significance in breast and other cancers. Given the essential role of antiviral pathways in immune surveillance, our mechanistic findings reveal previously unrecognized functions of pseudogenes and establish a paradigm in the regulation of antiviral and antitumor immunity.

Pseudogenes have long been regarded as nonfunctional genomic relics, primarily due to disruptive mutations or the absence of regulatory elements (1). However, the human genome harbors approximately 14,729 pseudogenes (2), a number comparable to that of protein-coding genes. These include pseudogenes derived from tumor suppressors and oncogenes such as *PTEN, KRAS*, *BRAF*, and *BRCA1* (3–7). Notably, pseudogenes have been shown to regulate their parental gene expression by controlling mRNA stability (8, 9), or acting as competitive endogenous RNAs (10). Increasing evidence however supports broader biological roles for pseudogenes, including modulation of immune responses and regulation of gene expression (1, 11).

Antiviral innate immune responses play a pivotal role in conferring intrinsic anticancer benefits by promoting antitumor immunity and enhancing the efficacy of chemotherapy and immunotherapy (12–15). These responses initiate key antitumor mechanisms, including the production of cytokines, activation of cytotoxic immune cells, and induction of cancer cell apoptosis. Intriguingly, recent studies indicate that innate antiviral immunity is regulated not only by viruses or exogenous nonself RNAs but also by host-derived endogenous RNAs, including transcripts from pseudogenes. For instance, 5S ribosomal RNA pseudogene transcripts (*RNA5SP141*) have been shown to bind RIG-I and induce antiviral cytokine expression during herpes simplex virus type 1 and Epstein–Barr virus infections (16). Additionally, the Lethe pseudogene (*Rps15a-ps4*) is selectively upregulated by proinflammatory cytokines or glucocorticoid receptor agonists and functions as a regulator of inflammatory signaling (17).

The accumulation or aberrant expression of endogenous RNAs in cancer can activate nucleic acid-sensing pattern recognition receptors (PRRs) such as RIG-I, MDA5, and PKR (18, 19). Engagement of these receptors triggers type I interferon (IFN) production and upregulation of interferon-stimulated genes (ISGs), leading to increased cytokine secretion, recruitment of cytotoxic immune cells, and apoptosis of malignant cells. Together, these findings indicate that pseudogene-derived endogenous RNAs can modulate antiviral innate immune responses and inflammatory pathways in human cells.

*BRCA1P1* is a partially duplicated pseudogene of *BRCA1* (Gene ID: 394269, HUGO ID: 28470), containing only three of the 24 exons present in the parental gene (20–22). Located on chromosome 17q21 adjacent to *BRCA1*, *BRCA1P1* is unique to higher primates, including humans, and is absent in rodents and chickens. Due to a point mutation in exon 1a that disrupts the translation initiation codon (ATG), along with additional degenerative mutations, *BRCA1P1* does not encode a protein. Instead, it is transcribed as a long noncoding RNA (lncRNA) localized to the nuclei of human cancer cells. Notably, *BRCA1P1* also harbors an insertion of the acidic ribosomal phosphoprotein P1 pseudogene (*RPLP1P4*) within exon 1a, giving rise to a chimeric pseudogene with sequences derived from both *BRCA1* and *RPLP1* (7).

Recently, we found that *BRCA1P1* functions as an immunoregulatory RNA that modulates antiviral immunity (7). In this study, we demonstrate that *BRCA1P1* transcripts exert pancancer immunoregulatory effects by suppressing antiviral responses not only in breast cancer cells but also in a variety of other cancer types. The majority of *BRCA1P1* transcripts are circular RNAs, highlighting the unique features of this pseudogene. We further assessed the therapeutic potential of targeting *BRCA1P1* in diverse cancer models, including patient-derived tumor organoids (PDOs) and a humanized mouse model. Data from these preclinical breast tumor models suggest that this host-derived pseudogene RNA has therapeutic potential for modulating innate antiviral immunity and antitumor immune responses and that these effects may extend to other tumor types.

## Results

### Pancancer Expression of *BRCA1P1*-lncRNA in Human Cancers.

**Fig. 1.**
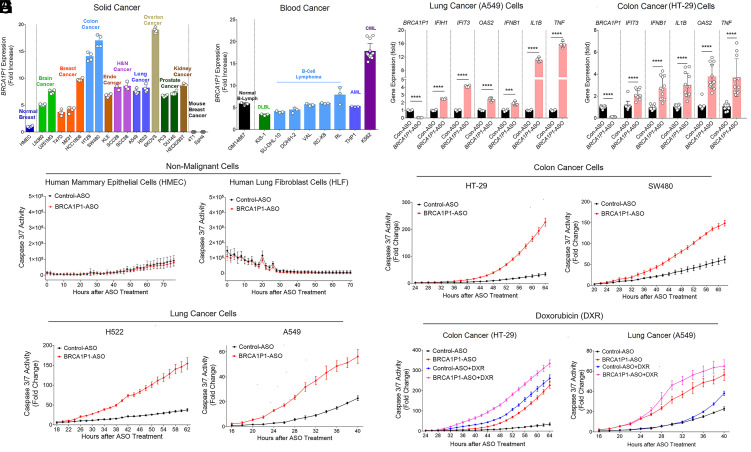
Increased antiviral gene expression and apoptosis in *BRCA1P1*-depleted pancancer cells. (*A* and *B*) *BRCA1P1* expression was examined in 28 cell lines using qRT-PCR analyses. Results were normalized to *RNA18S,* and the fold increase was calculated relative to the expression in human mammary epithelial cells (HMEC). Endo and H&N cancers represent endometrial and head and neck cancers, respectively. DLBL, diffuse large B-cell lymphoma; AML, acute myeloid leukemia; CML, chronic myeloid leukemia. (*C* and *D*) qRT-PCR analyses of antiviral gene expression in A549 (*C*) and HT-29 cells (*D*) transfected with control-ASO (con-ASO) or *BRCA1P1*-targeting ASO (*BRCA1P1*-ASO). Data represent the mean and SD of n > 3 biological replicates and are representative of at least two independent experiments. ***P < 0.001; ****P < 0.0001. (*E*–*H*) Apoptosis of *BRCA1P1*-ASO- and control-ASO-treated cells was analyzed using the IncuCyte Live-Cell Imaging System. Apoptosis was quantified in nonmalignant cells (*E*), colon cancer (*F*), and lung cancer cells (*G*) using green fluorescent signals from caspase-3/7-positive apoptotic cells normalized to cell density. Cancer cells were also treated with doxorubicin (DXR) (*H*). Data represent the mean and SD of n = 3 to 4 biological replicates and are representative of at least two independent experiments.

We previously identified *BRCA1P1*-lncRNA in the nuclei of human breast cancer cells (7). To investigate its expression across various cancer cells, we analyzed 26 different cell lines from 13 types of human cancers (brain, breast, colon, endometrial, head and neck, kidney, lung, prostate, ovarian cancers, DLBL, B cell lymphoma, AML, and CML). Interestingly, *BRCA1P1*-lncRNA was expressed at variable levels across cancer cell lines, with increased expression compared with human mammary epithelial cells (HMEC) (Fig. 1*A*). Notably, *BRCA1P1*-lncRNA was not expressed in mouse cancer cells, confirming its presence exclusively in the higher primate genome (including humans), but not in rodents. *BRCA1P1* was also detected at variable levels in hematologic malignancies (Fig. 1*B*). Overall, our data demonstrated ubiquitous expression of the *BRCA1P1*-lncRNA in all types of human cancers, with generally higher expression in cancer cells compared to normal cells.

### *BRCA1P1* Depletion Enhanced Antiviral Gene Expression across Multiple Cancer Types.

We next investigated whether *BRCA1P1* regulates the antiviral defense response across different cancer types, based on our previous observation that *BRCA1P1* inhibition led to innate antiviral gene expression in two breast cancer cell lines (MDA-MB-231 and T47D) (7). To suppress *BRCA1P1* expression in various cells, we synthesized 15-nt locked nucleic acid (LNA)-Gapmer antisense oligonucleotides (ASOs) with phosphorothioate linkages targeting the downstream junction of the *RPLP1P4* insert in exon 1a of *BRCA1P1*, without affecting the parent gene *BRCA1* expression. *BRCA1P1* knockdown resulted in increased expression of ISGs [*IFIH1* (MDA-5), *IFIT3*, and *OAS2*] as well as cytokine genes [*IFNB1* (IFN-β), *IL1B* (IL-1β), and *TNF* (TNF-α)] in A549 (lung cancer) cells (Fig. 1*C*). *BRCA1P1* depletion also stimulated antiviral gene expression in HT-29 (colon cancer), HCC1806 (breast cancer), and H522 (lung cancer) cells (Fig. 1*D* and *SI Appendix*, Fig. S1 *A* and *B*). The response to *BRCA1P1* knockdown varied among various cancer cells, with A549 cells exhibiting a dramatic increase in *TNF* expression (a 15.8 ± 0.9-fold increase), whereas HT-29 and HCC1806 cells displayed a more moderate increase (3.7 ± 1.6-fold and 2.9 ± 0.2-fold increases, respectively). These findings suggest that *BRCA1P1* depletion broadly activates antiviral responses, including cytokine and ISG production, although the magnitude of induction varies across cancer cell types.

### Depletion of *BRCA1P1* Increased Apoptosis and Drug Sensitivity in Pancancer Cells.

Host antiviral responses not only stimulate IFN and TNF cytokines but can also induce apoptosis in infected cells (23). Apoptosis of infected cells is a critical defense mechanism to limit the spread of viral infections. To determine whether *BRCA1P1* inhibition induces apoptosis in pancancer cells, we treated various cells with *BRCA1P1*-ASO. Depletion of *BRCA1P1* did not increase the number of caspase-3/7-positive apoptotic cells in nonmalignant cells, such as HMEC and human lung fibroblast cells (HLF) (Fig. 1*E*). In contrast, *BRCA1P1* depletion significantly increased apoptosis in colon (HT-29 and SW480), lung (A549 and H522), ovarian (SKOV3), and endometrial (KLE) cancer cells, as well as in triple-negative breast cancer (TNBC) cancer cells harboring either wild-type or mutant *BRCA1*, independently of *BRCA1* mutation status (Fig. 1 *F* and *G* and *SI Appendix*, Fig. S1 *C*–*E*). Furthermore, treatment with doxorubicin chemotherapy (DXR, Adriamycin) resulted in greater sensitivity in *BRCA1P1*-ASO-treated cancer cells compared to those treated with control-ASO (Fig. 1*H*), indicating that *BRCA1P1* depletion enhances the susceptibility of cancer cells to doxorubicin-induced apoptosis. Collectively, these findings suggest that *BRCA1P1* depletion effectively induces apoptosis in various types of cancer cells and enhances sensitivity to doxorubicin without exerting toxic effects on nonmalignant cells.

### Increased Sensitivity to Poly(I:C) in *BRCA1P1*-Knockout (KO) Cancer Cells.

**Fig. 2.**
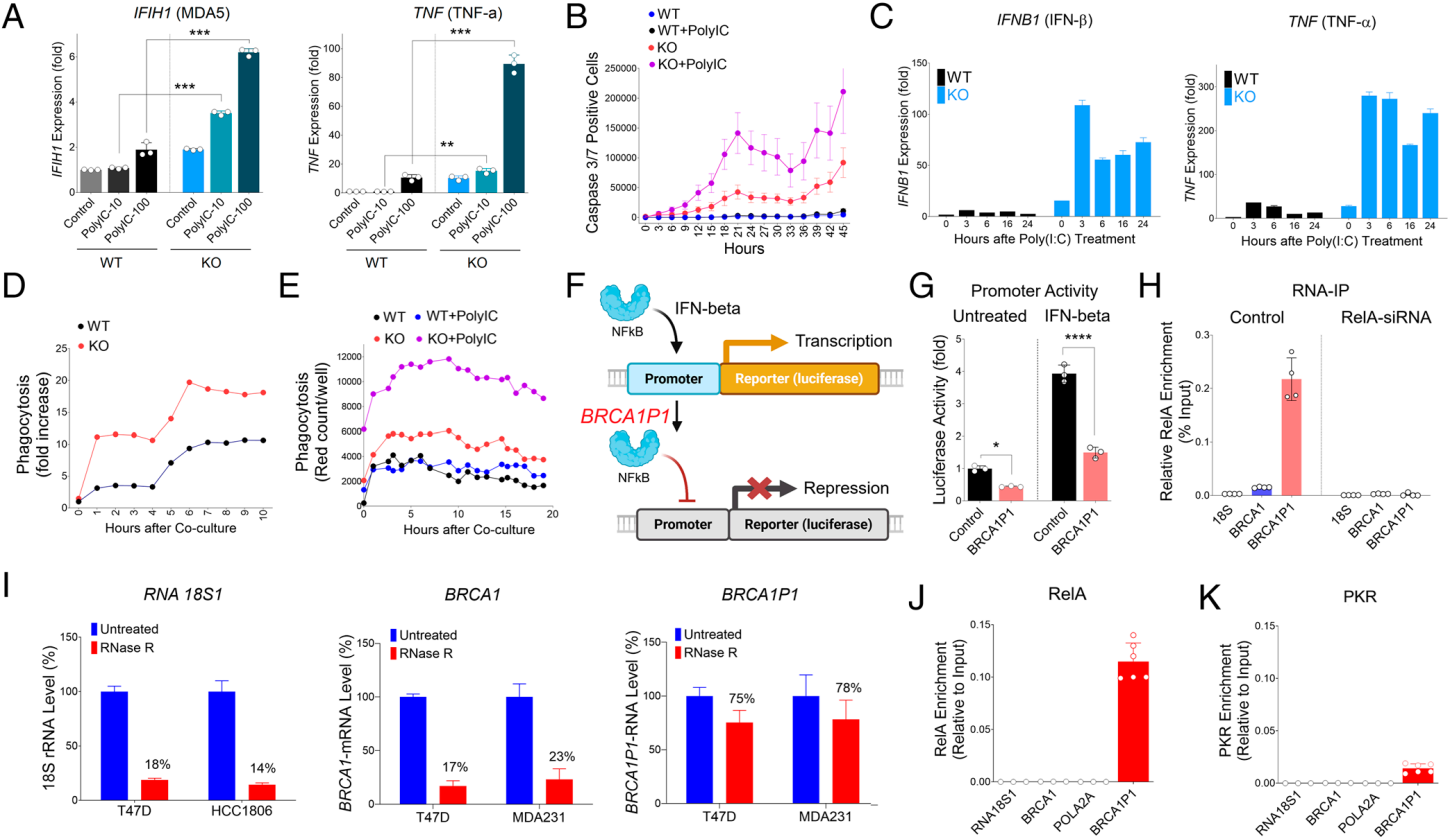
Increased Poly(I:C) sensitivity and phagocytosis in *BRCA1P1*-knockout cells, and regulation of NFκB activity by *BRCA1P1*. (*A*) qRT-PCR analysis of antiviral gene expression (*IFIH1* and *TNF*) in HT-29 cells with *BRCA1P1*-knockout (KO) or wild-type (WT) genotypes, with or without poly(I:C) stimulation at 10 or 100 μg. Data represent the mean and SD of n = 3 biological replicates and are representative of at least two independent experiments. **P < 0.01; ***P < 0.001. (*B*) Apoptosis of HT-29 cells with *BRCA1P1*-KO and WT genotypes was analyzed using the IncuCyte Live-Cell Imaging System with or without poly(I:C) treatment. (*C*) Time-dependent increases in antiviral gene expression (*IFNB1*and *TNF*) were measured by qRT-PCR at 3 to 24 h after treatment with 100 μg of poly(I:C). Data represent the mean and SD of n = 3 to 4 biological replicates. (*D* and *E*) HT-29-WT and *BRCA1P1*-KO cells were cocultured with M1-polarized THP-1 (*D*) or J774A.1 macrophages (*E*). Red-fluorescent cells per well were quantified in HT-29 cells stained with pHrodo Red using the IncuCyte® Live-Cell Analysis System, both in the absence and in the presence of poly(I:C). (*F*) Schematic representation of the reporter construct and the proposed model of *BRCA1P1*-mediated regulation of transcriptional activity. (*G*) Reporter activity assay in MDA-MB-231 cells transfected with the *IFIT3* promoter-luciferase construct in the presence or absence of in vitro transcribed *BRCA1P1*-RNA. One day after transfection, cells were treated with IFN-β and harvested for luciferase activity measurement. (*H*) RNA immunoprecipitation (RNA-IP) analysis in MDA-MB-231 cells using antibodies against the RelA transcription factor. Enrichment of *BRCA1P1* RNA is shown as a percentage of input RNA (% input), with 18S rRNA and *BRCA1* mRNA serving as negative controls. (*I*) Relative levels (%) of RNase R-resistant fractions of *RNA 18S1*, *BRCA1,* and *BRCA1P1* transcripts were examined in three breast cancer cells [T47D, HCC1806, and MDA-MB-231(MDA231)]. (*J* and *K*) RNA-binding assays were conducted on RNAase R-treated RNAs with RelA or PKR proteins. RelA or PKR enrichment on each RNA was quantified using qRT-PCR relative to input. Data represent the mean and SD of n = 3 biological replicates and are representative of at least two independent experiments.

Poly(I:C) (polyinosinic-polycytidylic acid) is a synthetic double-stranded RNA analog that mimics viral genetic material and is widely used to stimulate innate antiviral immune responses (24). To elucidate the role of *BRCA1P1* in regulating antiviral immunity in response to poly(I:C), we employed CRISPR-Cas9 genome editing to delete the *BRCA1P1* pseudogene in HT-29 colon cancer cells. Following the genetic modifications, we treated these engineered cells with two doses (10 or 100 μg) of poly(I:C). This resulted in dramatic increases in the expression of antiviral (*IFIH1*) and cytokine (*TNF*) genes in *BRCA1P1*-KO cells compared to wild-type HT-29 cells, in a dose-dependent manner (Fig. 2*A*). Accordingly, *BRCA1P1*-KO cells displayed elevated sensitivity to poly(I:C) treatment, which led to a significant increase in caspase-3/7-positive apoptosis compared to wild-type cells (Fig. 2*B* and *SI Appendix*, Fig. S2*A*).

Furthermore, we analyzed time-dependent increases in antiviral gene expression following poly(I:C) stimulation for durations ranging from 3 to 24 h. Interferon-β (*IFNB1*) and proinflammatory cytokines (*TNF* and *IL1A*) were rapidly induced at 3 h postpoly(I:C) treatment in *BRCA1P1*-KO cells (Fig. 2*C* and *SI Appendix*, Fig. S2*B*), whereas ISGs (*DDX58*, *IFIH1*, and *IFIT3*) and transcription factors (*STAT2* and *IRF1*) were gradually upregulated up to 24 h posttreatment (*SI Appendix*, Fig. S2 *C*–*E*). Notably, these increases were substantially higher in *BRCA1P1*-KO cells compared to wild-type cells, with a 107.8 ± 5.7 vs. 5.2 ± 0.6-fold increase for *IFNB1* and a 277.9 ± 10.5 vs. 30.5 ± 7.3-fold increase for *TNF* at 3 h postpoly(I:C) treatment (Fig. 2*C*). These data demonstrate that the loss of *BRCA1P1* significantly stimulates antiviral immune responses and enhances sensitivity to poly(I:C) treatment.

### Increased Phagocytosis of *BRCA1P1*-Depleted Cells Cocultured with Macrophages.

Cytokines induced by antiviral immunity can activate macrophages, subsequently leading to phagocytosis of cancer cells (25, 26). We thus examined whether antiviral responses activated by *BRCA1P1*-depletion stimulated macrophage-driven phagocytosis. HT-29 colon cancer cells were cocultured with M1-polarized macrophages from THP-1 or J774A.1 monocyte cells. The intensity of macrophage phagocytosis was measured in real time by quantifying pHrodo red-stained cells (Fig. 2*D* and *SI Appendix*, Fig. S2*F*). The loss of *BRCA1P1* enhanced the intensity of phagocytosis in *BRCA1P1*-KO cells compared to the wild-type HT-29 cells cocultured with THP-1 macrophages. Furthermore, treatment with poly(I:C) increased phagocytosis in *BRCA1P1*-KO cells cocultured with J774A.1 macrophages (Fig. 2*E*). Collectively, these results indicate that antiviral immunity induced by *BRCA1P1*-loss stimulates macrophage-mediated phagocytosis of cancer cells.

### Regulation of *IFIT3* Promoter Activity by *BRCA1P1*.

To elucidate the molecular mechanism of *BRCA1P1*-driven antiviral immunity and to determine whether *BRCA1P1* regulates antiviral gene transcription, we generated a reporter plasmid containing the *IFIT3* promoter upstream of a luciferase gene and assessed promoter activity following *BRCA1P1* overexpression. (Fig. 2*F*). The basal promoter activity was significantly reduced in *BRCA1P1*-overexpressing MDA-MB-231 cells compared to control cells (0.44 vs. 1.0; *Left* panel, Fig. 2*G*). Upon IFN-β treatment, luciferase activity increased by 3.93 ± 0.13-fold in control cells but only by 1.49 ± 0.13-fold in *BRCA1P1*-cotransfected cells (*Right* panel, Fig. 2*G*). These results indicate that *BRCA1P1* attenuates antiviral *IFIT3* gene expression, potentially by interfering with transcription factor activity regulating antiviral genes. Previously, we demonstrated a physical interaction between *BRCA1P1*-lncRNA and the NF-κB subunit RelA (p65) (7). To further verify this association, we performed RNA immunoprecipitation (RIP) assays in MDA-MB-231 cells following RelA siRNA treatment. *BRCA1P1*-RNA coprecipitated with RelA in control cells (*Left* panel, Fig. 2*H*), whereas RelA knockdown completely abolished this interaction (*Right* panel, Fig. 2*H*). Collectively, these findings suggest that *BRCA1P1* suppresses antiviral gene expression, at least in part, through a specific interaction with RelA proteins.

### The Majority of *BRCA1P1* Transcripts Are Circular RNAs.

Circular RNAs (circRNAs) are a class of endogenous single-stranded RNA molecules distinguished by their covalently closed loop structure, in which the 3′ and 5′ ends are joined together (27–29). Unlike linear RNAs, circRNAs lack free ends, rendering them highly resistant to exonuclease-mediated degradation and thus remarkably stable within cells. As circRNAs are stable in human cells and their expression is associated with various human diseases, we investigated whether *BRCA1P1* transcripts generate circRNAs in cancer cells. Breast cancer cells were treated with RNase R, an enzyme that selectively degrades linear RNAs while leaving circRNAs intact. Following RNase R treatment, 18S-rRNA and *BRCA1*-mRNA levels in two breast cancer cells were markedly reduced, with only 14 to 23% of transcripts remaining (*Left* and *Middle* panels, Fig. 2*I*). In contrast, the majority of *BRCA1P1* transcripts persisted after RNase R treatment, with 75.3 ± 11.3% and 78.2 ± 17.9% detected in T47D and MDA-MB-231 cells, respectively (*Right* panel, Fig. 2*I*). These results demonstrate a pronounced difference in RNase R resistance between the parental *BRCA1* and pseudogene *BRCA1P1* transcripts. The data indicate that most *BRCA1P1* transcripts (70 to 80%) exist as circRNAs, whereas only a small fraction (10 to 20%) of *BRCA1*-mRNA may form circular structures, possibly due to transient lariat intermediates generated during splicing.

As some endogenous circRNAs produced by mRNA splicing have been reported to interact with innate immune sensors (30), we also assessed potential binding to PKR. RNA binding assays confirmed that RelA strongly associated with *BRCA1P1* transcripts, with minimal or no binding observed for *18S rRNA*, *BRCA1*, or *POLA2A* transcripts (Fig. 2*J*). PKR exhibited a modest association with *BRCA1P1*-circRNAs, but this interaction was significantly weaker than that observed with RelA (Fig. 2*K*). To precisely map the sites of circRNA formation, we performed RT-PCR using divergent primers and identified circular junctions linking the 5′ and 3′ ends of *BRCA1P1* (*SI Appendix*, Fig. S2*G*). Sequencing of individual circRNA clones revealed at least five distinct circRNA isoforms, ranging from 753 to 1,160 nucleotides, composed of various combinations of the 5′ UTR, exon 1a, intron 1a, exon 1b, and intron 1b. Collectively, these findings demonstrate that *BRCA1P1* predominantly generates diverse circRNAs that are primarily associated with the RelA protein.

### RNA In Situ Hybridization (ISH) and Copy Number Analysis of *BRCA1P1* in Patient Tissues.

**Fig. 3.**
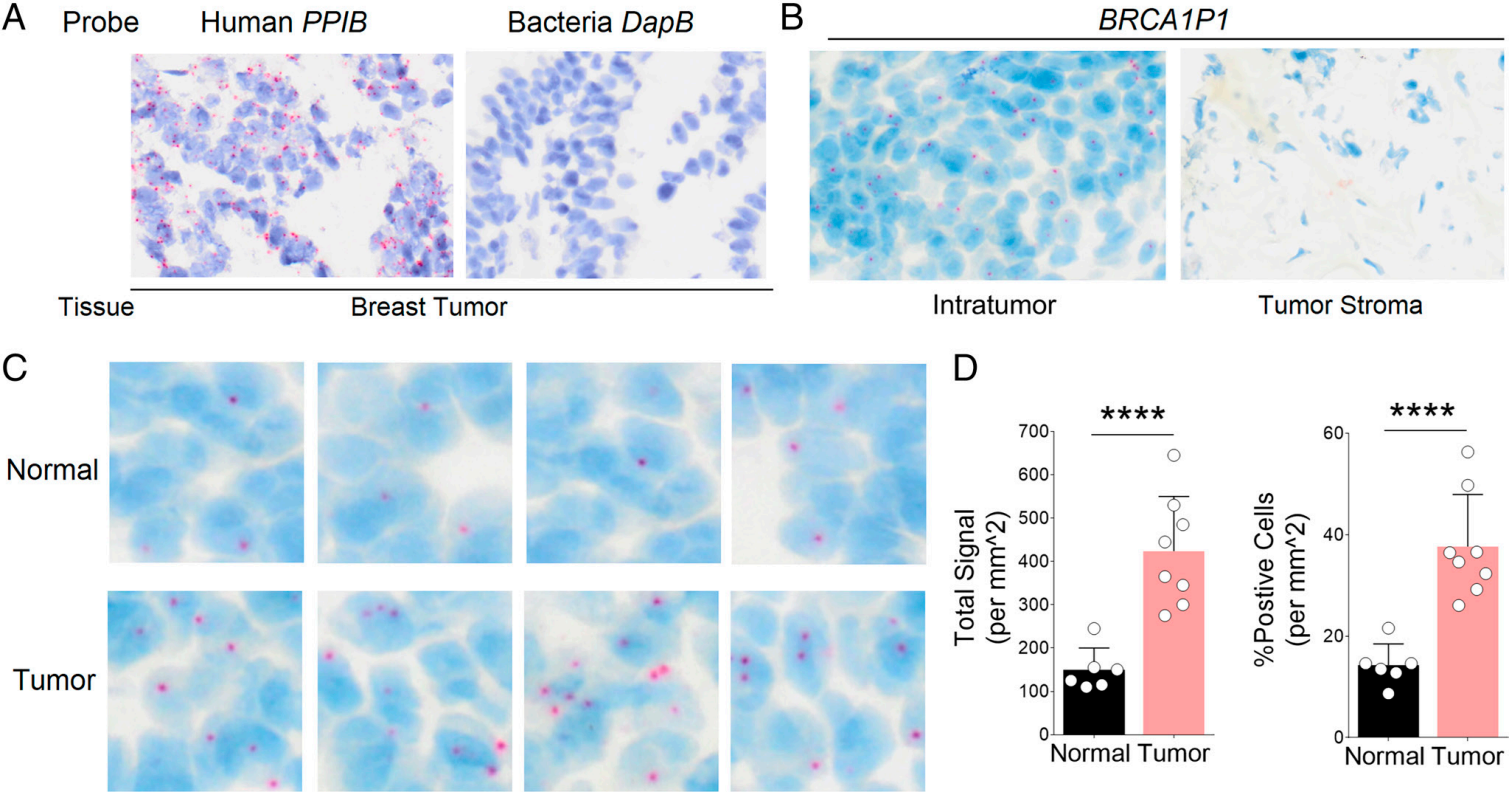
Increased *BRCA1P1* transcripts in breast tumors. Detection of *BRCA1P1*-RNA in human samples using BaseScope technology. (*A*) A positive control (human *PPIB*) and a negative control (bacterial *DapB*) were hybridized to fresh-frozen OCT-embedded breast tumors, exhibiting strong red signals and no signals, respectively. (*B*) A single ZZ-pair probe specific to *BRCA1P1* transcripts was hybridized to the intratumor region (not the stroma) of breast tumors. (*C*) Four representative images each from normal breast tissues (*Top*) and breast tumors (*Bottom*). (*D*) The total signals (*Left*) and percentage positive cells per mm^2^ (*Right*) of *BRCA1P1* transcripts were compared between normal breast tissues and breast tumors. ****P < 0.0001.

To assess the clinical relevance of *BRCA1P1* expression in patient tumors, we performed RNA-ISH on breast tissue specimens (*SI Appendix*, Table S1) using BaseScope technologies (Fig. 3). A single ZZ-pair probe was designed to specifically detect *BRCA1P1* and was experimentally validated in mouse xenograft tumors harboring either *BRCA1P1* wild-type or knockout genotypes (*SI Appendix*, Fig. S3*A*). *BRCA1P1* wild-type tumors displayed distinct red signals that were absent in knockout tumors, confirming specific detection of *BRCA1P1* transcripts without cross-reactivity to *BRCA1* transcripts. When breast tumors were hybridized with a positive control (human *PPIB*) and a negative control (bacterial *DapB*), the results showed strong red signals for the positive control and no signals for the negative control, respectively (Fig. 3*A*). Although the BaseScope assay is not specifically designed to distinguish nuclear from cytoplasmic localization, we interpreted signals overlapping with the DAPI-stained (blue) area as nuclear and observed that most *BRCA1P1* signals appeared within the DAPI-stained region, with only a small fraction detected outside this area (*SI Appendix*, Fig. S3*B*). Additionally, *BRCA1P1* transcripts were predominantly localized to the intratumoral region and were rarely detected in the tumor stroma (Fig. 3*B*). When comparing *BRCA1P1* signals between breast tumors and normal breast tissues, a significantly higher expression of *BRCA1P1* transcripts was observed in breast tumors (Fig. 3*C*). Both the total signals per mm^2^ and percent positive cells were markedly higher in breast tumors compared to normal breast tissues (Fig. 3*D*). The data indicate a significant increase in *BRCA1P1* expression in breast tumors relative to normal breast tissues, suggesting a potential role for *BRCA1P1* upregulation in breast tumors.

### *BRCA1P1*-Depletion Inhibited Viability of Patient-Derived Organoids of Breast Tumors.

**Fig. 4.**
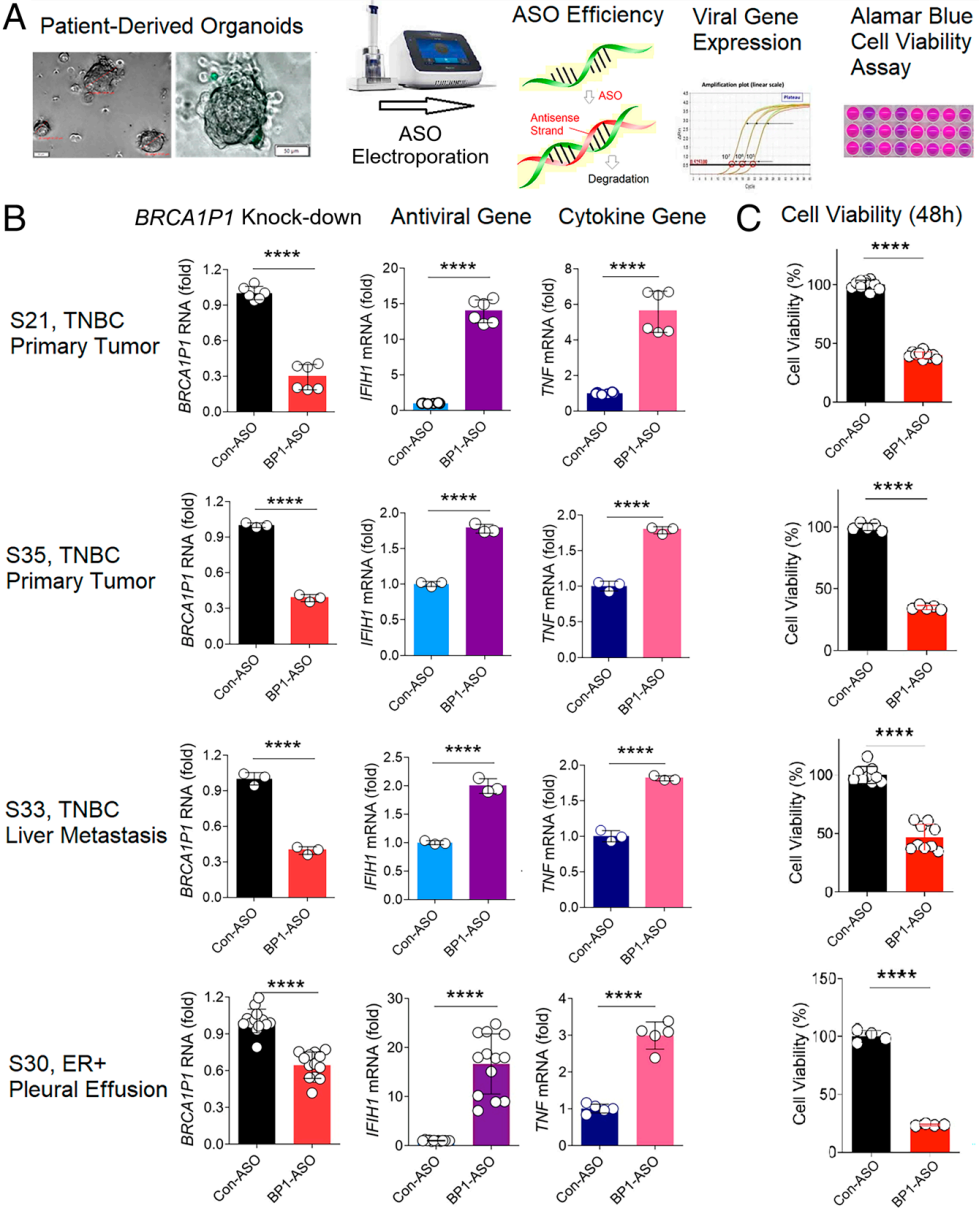
Reduced viability of PDOs with *BRCA1P1*-ASO. (*A*) PDOs were transfected with Control-ASO (Con-ASO) or *BRCA1P1*-ASO (BP1-ASO) using an electroporation system and subjected to qRT-PCR and cell viability assays. (*B*) The levels of *BRCA1P1*, MDA-5 (*IFIH1*), and TNF-α (*TNF*) transcripts were analyzed in BP1-ASO-treated PDOs relative to those in the Con-ASO-treated groups. (*C*) Viability of breast tumor PDOs was measured by Alamar blue assays and compared between the control and BP1-ASO-treated groups. All data represent the mean and SD of n = 3 to 12 biological replicates (****P < 0.0001).

To determine the therapeutic significance of *BRCA1P1*-inhibition in preclinical tumor models, we evaluated the antitumor efficacy of *BRCA1P1*-depletion in various PDOs from breast cancer patients at the University of Chicago Hospitals (31) (Fig. 4*A*). PDO S21 and S35 were derived from primary tumors of TNBC patients, whereas S33 and S30 were from liver metastasis of TNBC and lung metastasis of estrogen receptor (ER)-positive breast tumors, respectively (*SI Appendix*, Table S2). Electroporation of *BRCA1P1*-ASO significantly inhibited *BRCA1P1* expression in various PDOs, with 70%, 61%, 60%, and 35% knock-down efficiency in S21, S35, S33, and S30 PDOs, respectively (*Left*, Fig. 4*B*). Notably, depletion of *BRCA1P1* stimulated antiviral and cytokine gene expression, showing 13-fold and sixfold increases in *IFIH1* and *TNF* mRNA expression, respectively, in the *BRCA1P1*-ASO-treated S21 PDOs, compared to the control-ASO treated S21 PDOs (*Middle* and *Right*, Fig. 4*B*). More importantly, *BRCA1P1* inhibition significantly reduced breast tumor cell viability, resulting in a 53 to 76% decrease across *BRCA1P1*-ASO-treated PDOs (Fig. 4*C*). Collectively, our data demonstrated the regulation of antiviral/cytokine gene expression and tumor cell viability by *BRCA1P1* in preclinical tumor models of PDOs.

### The Loss of *BRCA1P1* Increased Immune Infiltration into Tumors in Humanized Mice.

We previously examined the physiological role of *BRCA1P1* in regulating tumor growth and antitumor immunity in athymic nude mice engrafted with MDA-MB-231 breast cancer cells with *BRCA1P1*-WT or KO genotypes (7). However, since these mice lack T cells, B cells, and NK cells, we were unable to determine the effect of *BRCA1P1*-KO on immune cell trafficking within these tumors. Furthermore, *BRCA1P1* knockout completely inhibited tumor growth in MDA-MB-231 tumors, preventing us from investigating the intratumoral effects of *BRCA1P1*-KO in this mouse model. Therefore, in this study, we utilized humanized NCG-B2m-KO mice with human Peripheral Blood Mononuclear Cells (PBMCs), as these mice express low levels of MHC I on cell membranes and are relatively resistant to graft-versus-host disease. HCC1806 breast cancer cells were selected for tumor xenograft experiments because *BRCA1P1*-KO produced only moderate TNF induction (2.0 ± 0.3-fold), intermediate apoptosis, and modest growth inhibition, whereas MDA-MB-231 cells exhibited a robust *TNF* response (12.6 ± 0.4-fold increase), marked apoptosis, and strong growth inhibition upon *BRCA1P1*-KO (*SI Appendix*, Fig. S4 *A*–*C*). This approach allowed us to investigate immune infiltration into tumors whose growth was moderately, rather than completely, inhibited by loss of *BRCA1P1*.

**Fig. 5.**
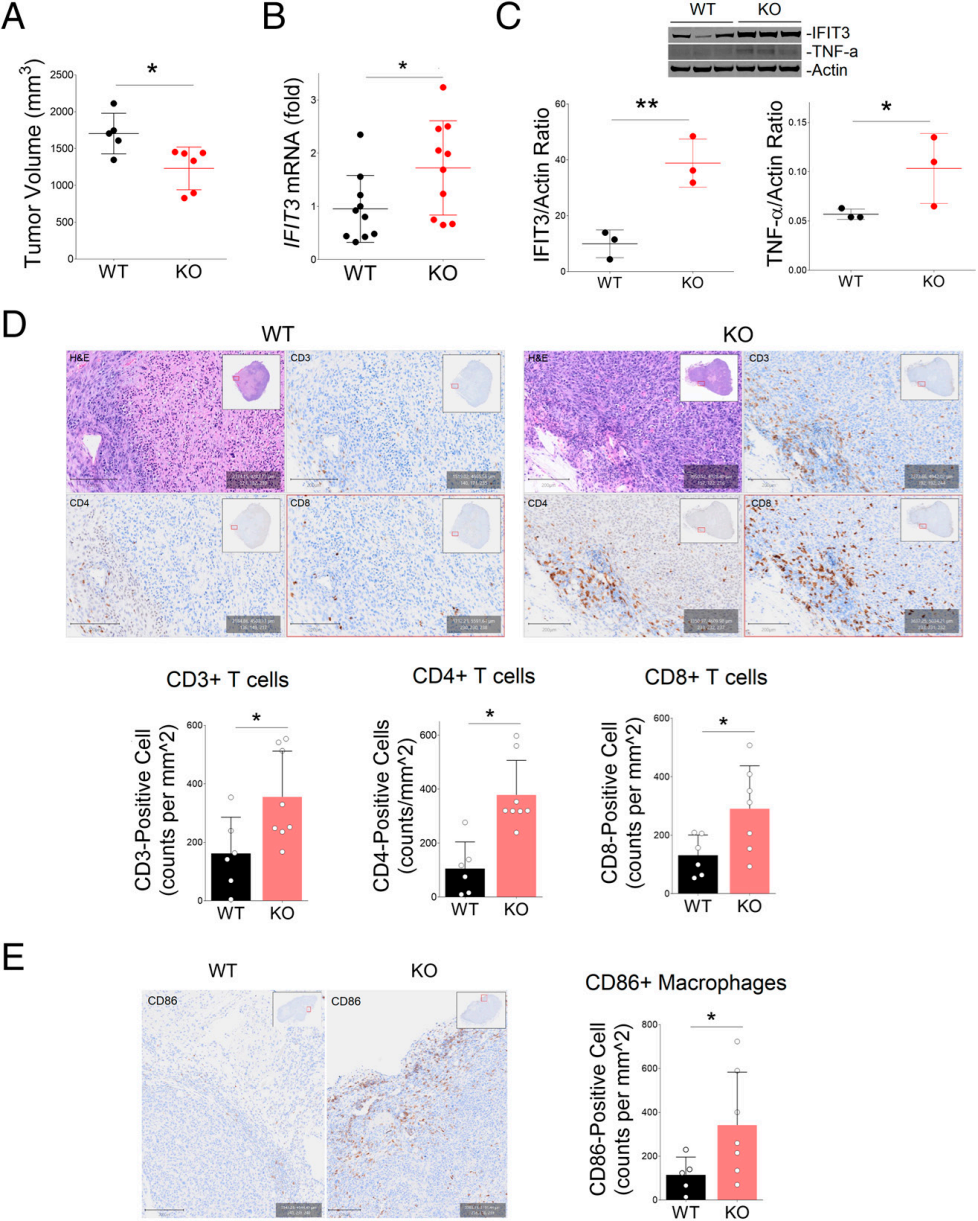
Reduced tumor volume and enhanced immune cell infiltration in *BRCA1P1*-KO tumors in humanized mice. NCG-B2m-KO mice were humanized with human PBMC and engrafted with HCC1806 breast cancer cells with *BRCA1P1*-WT or KO genotypes. (*A*) Tumor volume was compared between mice with *BRCA1P1*-KO and WT genotypes at the end of the study. The data represent the mean and SD of n = 5 to 6 mice (*P < 0.05). (*B* and *C*) Expression of antiviral gene (*IFIT3*) and proinflammatory cytokine TNF-α (*TNF*) was measured by qRT-PCR (*B*) and western blot analyses (*C*) in tumors collected at day 38 (*P < 0.05; **P < 0.01). (*D*) Representative images of H&E (*Top Left*), CD3 (*Top Right*), CD4 (*Bottom Left*), and CD8-positive (*Bottom Right*) cells infiltrated into *BRCA1P1*-WT and KO xenograft tumors (*Top*). Quantification of T cell infiltration in intratumoral regions of *BRCA1P1*-WT and KO xenograft tumors (*Bottom*). (*E*) Representative images (*Left*) and quantification (*Right*) of CD86+ macrophage infiltration in *BRCA1P1*-WT and KO xenograft tumors. The data represent the mean and SD of n = 5 to 8 mice (*P < 0.05).

NCG-B2m-KO mice were humanized with human PBMC and engrafted with HCC1806 breast cancer cells with *BRCA1P1*-WT or KO genotypes. (Fig. 5). Tumors were harvested at 38 d postinjection and subjected to immunohistochemistry (IHC), qRT-PCR, and western blot analyses. We observed a moderate difference in tumor growth between humanized mice engrafted with HCC1806 breast cancer cells carrying the *BRCA1P1*-KO or WT genotypes. The final tumor volume was significantly decreased in *BRCA1P1*-KO tumors compared to WT tumors (Fig. 5*A*).

When we examined antiviral gene expression in the tumors, we observed significant increases in IFIT3 and TNF-α transcripts and protein expression (Fig. 5 *B* and *C* and *SI Appendix*, Fig. S4*D*). The infiltration of human CD3-, CD4-, and CD8-positive T cells was moderately and significantly increased in intratumoral regions of *BRCA1P1*-KO tumors compared to the WT tumors (Fig. 5*D* and *SI Appendix*, Fig. S4*E*). A similar trend of increased infiltration in the stroma of *BRCA1P1*-KO tumors was observed (*SI Appendix*, Fig. S4*F*). Infiltration of CD86-positive (M1) macrophages was also significantly increased in *BRCA1P1*-KO tumors compared to WT tumors (Fig. 5*E*). Collectively, these data suggest that loss of *BRCA1P1* enhances antiviral gene expression and promotes immune cell infiltration into tumors, which could potentially contribute to reduced tumor growth in humanized mice.

## Discussion

**Fig. 6.**
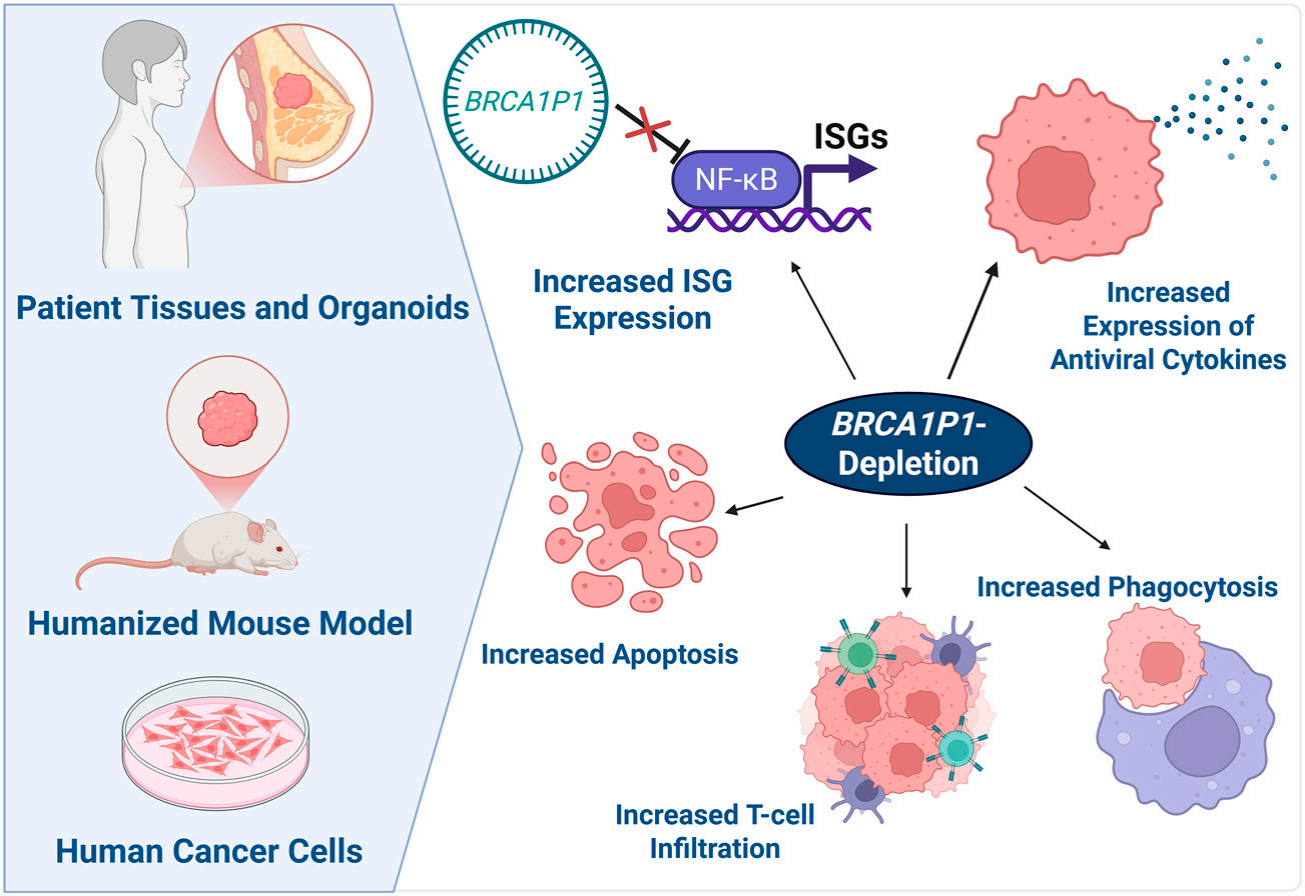
Proposed model of *BRCA1P1* regulation of antitumor immunity in preclinical breast cancer models. The functional role of *BRCA1P1* was investigated using breast tumor patient tissues and organoids, humanized mouse models, and pancancer human cell lines (*Left*). In *BRCA1P1* wild-type cells, *BRCA1P1*-circRNA binds to the NF-κB subunit RelA, suppressing NF-κB activity and thereby downregulating transcription of ISGs. Conversely, depletion of *BRCA1P1* allows NF-κB to occupy target promoters and promote ISG expression, which upregulates cytokine production and induces cancer cell apoptosis (*Right*). These events enhance macrophage-mediated phagocytosis and stimulate T lymphocyte infiltration within the tumor microenvironment.

Although pseudogenes are abundant in the human genome, with approximately 14,729 identified (1, 2), they have long been regarded as nonfunctional genomic relics. Recent research, however, has begun to reveal important roles for pseudogene RNAs in antiviral and antitumor defense mechanisms. In this study, we delineated the functional significance of *BRCA1P1* in human malignancies through comprehensive studies utilizing diverse pancancer human cell lines (Figs. 1 and 2), breast tumors and normal tissues (Fig. 3) and breast tumor organoids (Fig. 4), and humanized murine models (Fig. 5). Based on our findings, we propose a model for *BRCA1P1*-mediated regulation of antitumor immunity in preclinical cancer models (Fig. 6). In wild-type cells, *BRCA1P1*-circRNA interacts with the NF-κB subunit RelA, inhibiting NF-κB-mediated transcription of interferon-stimulated genes. In contrast, *BRCA1P1*-depletion facilitates NF-κB recruitment to target gene promoters, thereby stimulating ISG expression. Subsequently, these molecular events potentiate macrophage-mediated phagocytosis and augment T lymphocyte infiltration within the tumor microenvironment. Collectively, these data suggest that *BRCA1P1* functions as an immunosuppressive RNA, and its inhibition activates antiviral defense pathways and enhances antitumor immune responses.

It is noteworthy that *BRCA1P1* is a fusion pseudogene derived from the *BRCA1* and *RPLP1* genes and contains two inserted ALU elements (*SI Appendix*, Fig. S5*A*). Although its name suggests similarity to BRCA1, a key regulator of DNA damage repair, *BRCA1P1* functions as a noncoding circRNA with distinct properties, in part because it incorporates *RPLP1* pseudogene sequences and ALU elements. Consistent with this, we observed no significant association between *BRCA1P1* expression and *BRCA1/2* somatic mutations in TCGA breast and ovarian tumor datasets or in breast cancer cell lines (*SI Appendix*, Fig. S5*B*). *BRCA1P1* copy number also did not differ between *BRCA1* wild-type and mutant breast or ovarian tumors (*SI Appendix*, Fig. S5*C*), nor between *TP53* wild-type and mutant diffuse large B-cell lymphomas (*SI Appendix*, Fig. S5*E*). Furthermore, no significant differences in *BRCA1P1* expression were observed between *TP53*-mutant and wild-type blood cancer cell groups (*SI Appendix*, Fig. S5*D*). Experimentally, *BRCA1P1* depletion increased apoptosis and camptothecin sensitivity in both *BRCA1*-mutant and wild-type TNBC cells, with heterogeneous responses across cell lines, independent of *BRCA1* mutation status (*SI Appendix*, Fig. S6). Collectively, these data indicate that neither *BRCA1P1* expression nor copy number correlates with *BRCA1* or *TP53* mutation status and that TNBC cell lines display heterogeneous responses to *BRCA1P1* depletion irrespective of *BRCA1* status. These findings suggest that *BRCA1P1* is unlikely to primarily regulate DNA damage repair pathways but instead plays a distinct role in modulating antiviral innate immune responses in breast and other cancers.

CircRNAs can originate from viral RNA genomes or from the processing of precursor messenger RNAs (pre-mRNAs) and noncoding RNAs (32). Recent studies suggest that circRNAs are involved in regulating innate immune responses (33). The emerging use of synthetic RNA circles to modulate cellular processes, influence immune responses, and to enable protein translation is opening new avenues in biomedical research (34). Notably, *BRCA1P1* transcripts predominantly form circular RNAs. More than 75% of *BRCA1P1* transcripts remain after RNase R treatment in both cell lines examined, suggesting a high level of circRNA formation. The remaining ~25% of *BRCA1P1* transcripts are likely linear RNAs that have not undergone circularization and therefore remain sensitive to RNase R. At least five distinct circRNA isoforms were identified in *BRCA1P1* transcripts, demonstrating the diversity of circularization events. The circular structure of *BRCA1P1* transcripts confers greater stability compared to linear RNAs, which makes them attractive candidates for biomarker development and therapeutic applications (33), suggesting that *BRCA1P1*-circRNAs may be developed as biomarkers and potential therapeutic targets in cancer.

Although our initial *BRCA1P1*-targeting ASOs have shown promising results in patient-derived organoids, further advances in ASO chemistry and formulation are needed to improve preclinical efficacy. To address these limitations, we plan to employ chemical modifications and optimize ASO delivery by leveraging cancer metabolism and developing cancer-targeted nanoparticles for enhanced delivery to solid tumors (35). Understanding these factors will be critical for the development of more effective cancer treatments targeting *BRCA1P1*.

We observe cellular heterogeneity in the response to *BRCA1P1* depletion between MDA-MB-231 and HCC1806 cells, despite their similar basal *BRCA1P1* expression levels. As reported previously, *BRCA1P1* depletion markedly stimulated *TNF* expression, strongly induced apoptosis, and almost completely inhibited MDA-MB-231 tumor growth (7), whereas in HCC1806 tumors, it only moderately increased *TNF* expression, induced intermediate levels of apoptosis, and partially inhibited tumor growth. As expected, *BRCA1P1*-KO in HCC1806 xenografts results in only a moderate reduction in tumor growth, allowing the collection of residual tumors for IHC analysis of immune infiltration.

In summary, we have identified a mechanism of innate immunity regulated by a pseudogene in pancancers. *BRCA1P1* is located at chromosome 17q21, near major tumor suppressors and oncogenes associated with genomic instability. Notably, the adjacency of *BRCA1* and *BRCA1P1* creates a hotspot for homologous recombination, and genomic rearrangements between these loci have been observed in families at high risk of breast and ovarian cancers (22). Furthermore, chromosome 17q abnormalities, primarily gains and structural rearrangements, are common in several cancers, including neuroblastoma, breast cancer, and various leukemias (36, 37). While previous studies have extensively mapped chromosome 17q21 in relation to aggressive breast cancer, they have primarily focused on protein-coding genes, with noncoding genes largely overlooked. Considering the importance of this genomic region across different cancers and the stability of *BRCA1P1*-derived circular RNAs, which are resistant to exonuclease-mediated degradation, our work may uncover a mechanism regulating antiviral and antitumor immune responses through host-derived pseudogene transcripts. The finding of BRCA1P1’s involvement in immune regulation has clinical implications for developing cancer interception and treatment strategies and warrants further investigation.

## Materials and Methods

### Animal Study.

All animals were humanely handled and monitored for health in accordance with Institutional Animal Care and Use Committee (IACUC)- approved protocols. Six to seven-week-old female NCG-B2m-KO mice (Charles River Laboratories) were anesthetized via inhalation with 2% vaporized isoflurane and unilaterally injected with 3 to 5.5 × 10^6^ HCC1806 cells with *BRCA1P1*-KO or WT genotypes (100 µL, 50% Matrigel, 6 to 10 animals per group) into the fourth inguinal mammary gland at the base of the nipple. Mice were also injected with 1 × 10^7^ human PBMCs using the PBMC Select Humanization Kit (PB009C-50-CRL) via tail vein injection. Animal body weights were measured three times, and general health status was monitored. Tumor measurements were performed weekly using calipers to calculate tumor volume using the formulas: 1/2 (Length × Width^2^) or the ellipsoid volume formula (3/4 πabc, the semiaxes of the ellipsoid) after tumor isolation.

### Mouse Tumor Processing and Data Analyses.

Tumors were collected from animals in each model at the end of the study for downstream experiments. RNA and proteins were extracted from snap-frozen tumors using the RNeasy Mini Kit (Qiagen #74104) and RIPA Buffer (Thermo Fisher Scientific #89900) according to the respective vendor’s protocols and subjected to qRT-PCR and Western Blot analyses. 15 to 30 mg of frozen tumor tissue was cut out on dry ice and homogenized by TissueLyser LT, programmed to run at 40 Hz for 1 min, to reach high RNA or protein yield. Formalin-Fixed Paraffin-Embedded (FFPE) tumor tissues were cut to slides then subjected to IHC against CD3 (abcam #ab16669, 1:100 dilution), CD4 (Thermo Fisher Scientific #MA5-16338, 1:50 dilution), CD8 (Thermo Fisher Scientific #MA5-14548, 1:200 dilution), CD86 (Cell Signaling #91882, 1:100 dilution), and CD206 (Cell Signaling #24595, 1:1,000 dilution). IHC was conducted and analyzed as previously described (38). Briefly, scoring was performed independently and quantitatively by one experienced pathologist (GFK) and one researcher (YJH). The density (number) of positive cells was estimated by counting all immunopositive cells in five high-powered fields (0.85 mm^2^ each). The mean score was calculated by an average score assigned by a pathologist and a researcher. The result was presented as the number of positively stained cells per 1 mm^2^.

### Statistical Analysis.

Each experiment was conducted with n = 3 to 10 biological replicates and repeated at least twice. The mean ± SD is shown with *t* test (unpaired, two-tailed) values. Statistical significance was set at *P < 0.05, **P < 0.01, ***P < 0.001, and ****P < 0.0001. A one-way ANOVA was performed to compare expression levels across breast tumor subtypes. Statistical analysis was carried out using Microsoft Excel and GraphPad Prism 6.0 (GraphPad Software, Inc., La Jolla, CA). Plots were generated using GraphPad Prism 6.0.

Detailed methods are in the *SI Appendix, Methods*.

## Supplementary Material

Appendix 01 (PDF)

## Data Availability

Code used for TCGA and DepMap data analyses is available in a publicly accessible GitHub repository: https://github.com/olopade-lab/BP1 (39). Original Western blot images and CRISPR gRNA/ASO sequences are provided in *SI Appendix*. All other data are included in the article and/or *SI Appendix*.
