# Supplementary material for: Regulation of antiviral and antitumor immunity by the BRCA1 pseudogene in human cancers
Source: Proc Natl Acad Sci U S A. 2026 May 4;123(19):e2528911123. doi: 10.1073/pnas.2528911123 (PMC13167737; doi:10.1073/pnas.2528911123)
Supplement: Supplementary file 1 — Appendix 01 (PDF) [file pnas.2528911123.sapp.pdf]

Supplementary Information

Supplementary Methods

Cell Culture. Primary human mammary epithelial cells (HMEC) were purchased from Lonza (Allendale, NJ). Cancer cells were obtained from the American Type Culture Collection (ATCC, Manassas, VA) or the Cellular Screening Center at the University of Chicago. Cells were authenticated for species and unique DNA profile using short tandem repeat (STR) analysis by the provider (ATCC). Cells were cultured in media recommended by ATCC and tested negative for mycoplasma contamination using the MycoAlert Kit (Lonza # LT07-318).

Cell Transfection and IncuCyte Live-Cell Analysis. Cells were transfected with 20-50 nM of *BRCA1P1*-ASO (LNA GapmeRs, Exiqon, Denmark), as described previously (1). IncuCyte Caspase-3/7 Green reagent was added to the media at a 1:1000 dilution (Sartorius #4440). Images of live cells were acquired using a 10x objective every 2 hours (four images/well) with the IncuCyte® S3 Live-Cell Analysis System. Data were analyzed using IncuCyte analysis software. Cell density was quantified as a measure of cell proliferation. Apoptosis was quantified as the total integrated intensity of the green fluorescent signal from cells with activated Caspase-3/7 (apoptotic cells) normalized to cell density calculated from phase-contrast images. The experiment was performed at least twice with 3-8 technical replicates.

BaseScope RNA *In Situ* Imaging on Breast Tissues. All studies included in RNA-ISH were approved by the BSD/UCMC Institutional Review Boards at the University of Chicago (16352A). All participants in this study provided written informed consent to allow the use of their tissue samples for research. All methods were carried out in accordance with the guidelines and regulations of the University of Chicago. Written informed consent was obtained from the patients for the anonymous use of banked tumor tissues for research. The studies were conducted in accordance with the recognized ethical guidelines of the Declaration of Helsinki. Methods for case selection were described previously (1, 2). Fresh-frozen OCT-embedded tumors (n=8) and normal tissues (n=6) were hybridized with one ZZ pair probe specific to *BRCA1P1* using the BaseScope LS detection reagent kit (for use with Leica Biosystems' BOND RX System, ACD #323600). Human-PPIB-1ZZ (ACD #701078) and bacterial DapB-1ZZ probes (ACD #701028) were used as positive and negative controls, respectively. Scoring and calculation of the average score were performed by a pathologist (GFK) using the QuPath program, as previously described (3). The result was presented as the number of positively stained cells per mm^2

or percent positive cells per mm^2.

CRISPR-Cas9 Knockout. One pair of crRNAs was designed to delete 1,096 bp of the *BRCA1P1* genomic region using the IDT Alt-R CRISPR-Cas9 guide RNA tools. crRNAs were synthesized, annealed with tracrRNAs, and integrated with Alt-R S.p. Cas9 nuclease (IDT #1081058). HT-29 and HCC1806 cells were transfected with the RNP complex using Lipofectamine CRISPRMAX Cas9 transfection reagent (Thermo Fisher Scientific # CMAX00001) and incubated for two days. Single colony cells were isolated, expanded, and subjected to PCR validation and DNA sequencing, as described previously (1). The Alt-R CRISPR-Cas9 crRNA sequences are listed below.

**crRNA**	**Sequence**
*BRCA1P1*_707	/AlTR1/rUrGrUrUrGrArCrArUrGrUrArUrArArGrCrCrCrGrGrUrUrUrUrArGrArGrCrUrArUrGrCrU/AlTR2/
*BRCA1P1*_1830	/AlTR1/rArGrArUrGrGrGrUrArUrUrCrUrUrArUrGrCrGrArGrUrUrUrUrArGrArGrCrUrArUrGrCrU/AlTR2/

Breast Cancer PDO Development. The procurement of biospecimens for the generation of breast cancer PDOs was performed according to the approved institution review board protocol (16352A) with signed patient consents. All patient samples and the clinical information related to them were deidentified. Fresh core needle biopsy samples or cells from pleural effusions were obtained as remnant specimens without compromising necessary diagnostic procedures. Breast tissue from core biopsies was enzymatically digested for 1 hour to generate a cell suspension. Cells from pleural effusions were collected by centrifugation at 450g for 10 minutes and washed in Advanced DMEM-F12 (Thermo Fisher Scientific #12634010) supplemented with 10 mM Hepes (Thermo Fisher Scientific #15630080), 1X GlutaMAX (Thermo Fisher Scientific #35050061), and 1X Antibiotic-Antimycotic (Thermo Fisher Scientific #5240062) (AdvM+). The resulting cell pellets were mixed with Matrigel (Corning #356237), plated in prewarmed 24-well plates (Greiner Bio-One #662102) to allow Matrigel to polymerize at 37°C, and cultured in breast cancer PDO media to support organoid growth. Breast cancer PDO medium consists of AdvM+ medium supplemented with Y27632 (5 µM; AbMole #M20999), R-Spondin-3 (250 ng/mL; Thermo Fisher Scientific #120-44), Noggin (100 ng/mL; Thermo Fisher Scientific #120-10C), B27 (1X; Thermo Fisher Scientific #17504044), NIC (5 mM; Sigma #N0636-100G), NAC (1.25 mM; Sigma #A9165-5G), Primocin (100 µg/mL; Invitrogen #ant-pm-1), EGF (5 ng/mL; Thermo Fisher Scientific #AF-100-15), FGF-10 (20 ng/mL;

Thermo Fisher Scientific #100-26), A83-01 (500 nM; Tocris #2939), FGF-7 (5 ng/mL; Thermo Fisher Scientific #100-19), SB202190 (500 nM; Sigma #S7067), and Heregulin beta-1 (5 nM; Thermo Fisher Scientific #100-03). To prevent misidentification and cross-contamination, each PDO sample was cultured in a separate, uniquely labeled dish. Identity of PDOs was confirmed by DNA fingerprinting using the AmpFLSTR® Identifiler® PCR Amplification Kit (Thermo Fisher Scientific #4322288). All PDOs were tested for mycoplasma contamination and maintained at 37°C with 5% CO_2 unless otherwise indicated.

Electroporation of PDOs with ASO. Once organoids reached a size of 50 to 100 µm, they were harvested for subsequent experimental applications. To dissociate from Matrigel, PDOs were incubated with Dispase (STEMCELL Technologies #07913) at 37°C for 20 minutes, washed twice with AdvM+, and centrifuged at 450g for 5 minutes. To obtain a single-cell suspension, the PDO pellet was incubated with TrypLE (Thermo Fisher Scientific #12563011) at 37°C for 5 to 10 minutes. A 28G syringe was used to help disrupt cells by loading the TrypLE-PDO mixture in and out 5-7 times if PDO dissociation was not complete. Trypsinization was stopped with 10% FBS, and cells were washed twice with AdvM+ and centrifuged at 450g for 5 minutes before cell counting. Cells were mixed with 10 µl Neon Resuspension Buffer R and 150 pmol of ASO before electroporation using 1600V, 2 pulses, 20 msec/pulse by Neon NxT. These cells were added to 500 µl of prewarmed AdvM+ medium and incubated at 37°C for 30 minutes to allow recovery. Cells were then pelleted, and the medium was cleared until only 5 µl remained. Cells were mixed by pipetting, then 50-150 µl of Matrigel was added to the cells, depending on the cell number, and the mixture was mixed well, avoiding bubbles. The cell-Matrigel mixture were plated in prewarmed 96-well plates or 24-well plates, respectively. Plates were incubated at 37°C for 10 minutes (96-well plate) or 30 minutes (24-well plate). Prewarmed organoid culture medium, 100 µl for 96-well plates and 500 µl for 24-well plates, was added to the plate with solidified Matrigel cell domes. ASO sequences targeting *BRCA1P1* are disclosed in the pending international patent application WO2024006994A3 ("Compositions and methods for the treatment of cancer by targeting the BRCA1 pseudogene 1"; The University of Chicago). The full sequences can be accessed through the patent document.

Total RNA Extraction and qRT-PCR. Cells were transfected with 20-50 nM of control or *BRCA1P1*-ASO

(LNA GapmeRs, Exiqon, Denmark) as described previously (1) and processed for RNA purification using the RNeasy kit (Qiagen #74104) following the protocol provided by the manufacturer. To avoid any possibility of DNA contamination, total RNAs were treated with RNase-free DNase I (Qiagen #79254). One microgram of RNA per sample was used for cDNA synthesis using the SuperScript III First-Strand Synthesis kit (Thermo Fisher Scientific #11752050). qRT-PCR was performed using *BRCA1P1* primers and primers for other genes of interest with the Power SYBR Green (Thermo Fisher Scientific #4367659) or TaqMan Gene Expression Master Mix (Thermo Fisher Scientific #4369016). The fold change in expression of each gene was calculated using the ΔΔCT method, with 18S rRNA (RNA18S) as an internal control.

Protein Extraction and Western Blot. For total protein extraction, cells were lysed with Pierce RIPA buffer (Thermo Fisher Scientific #89900), sonicated for 10 seconds, and centrifuged for 15 minutes at 14,000g to collect the supernatant. An equal amount of protein for each lysate was analyzed by Bolt Bis-Tris Plus Gels (Thermo Fisher Scientific #NW04122BOX) and transferred to PVDF membrane (Thermo Fisher Scientific #IB24001) using the iBlot 2 Dry Blotting System. The membrane was blocked in Intercept Blocking Buffer (LI-COR #927-60001) and incubated with the primary antibody of interest at the recommended concentration at 4°C overnight, followed by incubation with a species-appropriate fluorescence-labeled secondary antibody (1:10,000 dilution) for 1 hour at room temperature. Membranes were scanned using LI-COR Odyssey M and data was analyzed using ImageStudio. The antibodies used are IFIT3 (B-7) (1:200 dilution, SantaCruz #sc-393512), TNFa (D5G9) (1:200 dilution, Cell Signaling #6945), and Actin (1:10,000 dilution, abcam #ab8227). The original Western blot image for Fig. 5C is provided in Supplementary Fig. S7.

Reporter Gene Activity Assays. Luciferase reporter gene assays were performed as previously described (1, 4). Briefly, HCC1806 cells were plated in 96-well plates and transfected with either the empty pGL3B vector or the pGL3B vector containing the IFIT3 promoter (25 ng per well). Truncated *BRCA1P1*-lncRNAs were *in vitro* transcribed using the HiScribe T7 high yield RNA synthesis kit (NEB #E2040) with pseudo-UTP (TriLink #N1081), and co-transfected into the cells (14 fmol per well). Twenty-four hours post-transfection, cells were treated with 250 U/mL of IFN-β (PBL Assay Science #11415-1) for an additional 24 hours, then harvested for

luciferase activity assays (Promega #E6110).

Phagocytosis Assays. THP1 monocytes were seeded at $1x10^5$ cells/well in a 24-well plate and differentiated into macrophages using 100nM PMA (Phorbol 12-myristate 13-acetate) (Sigma #P1585) for three days. They were then incubated with 20 ng/ml IFN-gamma (abcam #ab9659) and 5 µg/ml LPS (Thermo Fisher Scientific #00-4976-03) for one day. J774A.1 monocytes were seeded at $3x10^4$ cells/well in a 24-well plate and stimulated with 5µg/ml LPS for one day. HT-29 colon cancer cells ($5x10^4$ cells/well) with *BRCA1P1*-WT or KO genotypes were stained (30 mins staining time) with 100 nM or 500 nM pHrodo Red (Thermo Fisher Scientific #P36600) and added to plates containing macrophage cells to start co-culture. Images of live cells were acquired using a 20x objective every hour (nine images/well) with the IncuCyte® S3 Live-Cell Analysis System. Red-fluorescence signals were captured and analyzed using IncuCyte analysis software.

RNase R Treatment, RNA Binding Assays, and RNA Immunoprecipitation (RIP). Total RNA (10 µg) extracted from T47D and MDA-MB-231 cells was incubated with RNase R (NEB #M0100S), following the manufacturer's protocol. RNA binding assays were performed based on previously described methods (5) with minor modifications. Briefly, 1 µg of RNase R-resistant RNA was incubated with recombinant RelA (Active Motif #81086 or #31302) or PKR (Creative Biomart #EIF2AK2-138H) proteins. RNA-protein complexes were immunoprecipitated using anti-His-tag (Cell Signaling #12698) or anti-Flag-tag (Sigma #F3165) antibodies, followed by capture with protein A agarose beads (NEB #S1425S). The enrichment of RelA or PKR proteins was quantified by qRT-PCR and analyzed relative to input controls. RIP was performed according to the native RIP protocol as described (1) (2).

TCGA Analyses of *BRCA1P1* Expression and Copy Number. RNA expression analyses were performed in R using TCGA Pan-Cancer Atlas datasets for breast invasive carcinoma (TCGA-BRCA) and ovarian serous cystadenocarcinoma (TCGA-OV). Pseudogene-level expression values were obtained from RPKM matrices generated by Han *et al*. (6), who developed an RNA-seq processing pipeline specifically optimized to quantify

pseudogene expression from TCGA RNA-seq data; these matrices (Synapse syn1732077) contain pseudogene annotations that are not captured by standard TCGA gene-level pipelines and were therefore used as preprocessed inputs for the pseudogene-focused analyses in this study. *BRCA1* and *BRCA2* mutation status was derived from TCGA Pan-Cancer Atlas somatic mutation calls accessed via cBioPortal, with samples mapped to TCGA patient barcodes and classified as *BRCA1/2* wild type or *BRCA1/2* mutant. Two annotated transcripts, ENSG00000267340 (RP11-242D8.3) and ENSG00000267595 (RP11-242D8.2), were identified from the *BRCA1P1* exon 1a and exon 2 regions, respectively. *BRCA1P1* expression was approximated using these annotated transcripts, with a particular focus on ENSG00000267340, which lies within the experimentally validated transcriptional region of the *BRCA1P1* pseudogene defined by primer extension of the full-length *BRCA1P1* transcript (1,648 nt). For each dataset, *BRCA1P1* expression was quantified by extracting RPKM values from the RPKM matrices and aggregating expression at the patient level, whereas *BRCA1* mRNA, as a protein-coding gene, was analyzed separately using TCGA RSEM-normalized gene-level expression data processed with the same patient-level aggregation strategy. Expression distributions were visualized as boxplots with overlaid patient-level points, and differences between the *BRCA1/2* mutant and wild-type groups were assessed using the Kruskal–Wallis or two-sided Wilcoxon rank-sum tests.

Supplementary Figures and Figure Legends

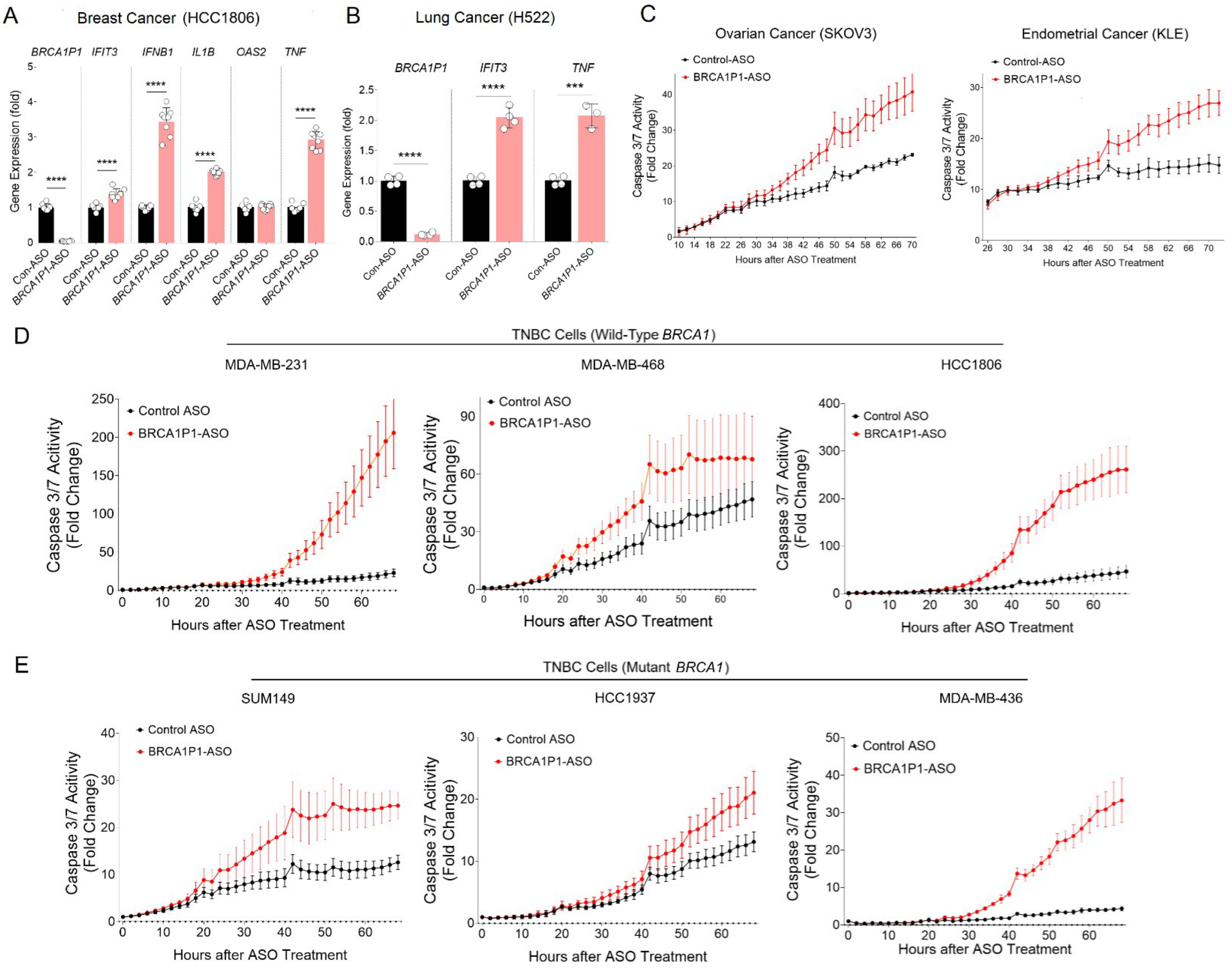

Supplementary Fig. S1. Increased Antiviral Gene Expression and Apoptosis in *BRCA1P1*-Depleted Cells. A and B. qRT-PCR analyses of antiviral gene expression in HCC1806 (A) and H522 cells (B) transfected with control-ASO (Con-ASO) or *BRCA1P1*-ASO. Data represent the mean and SD of n > 3 biological replicates and are representative of at least two independent experiments. ****, P < 0.0001. **C-E**. Apoptosis of *BRCA1P1*-ASO and control-ASO treated cells was evaluated in ovarian and endometrial cancer cells (C), as well as in TNBC cells harboring either wild-type (D) or mutant *BRCA1* (E), using the IncuCyte Live-Cell Imaging System. Apoptosis was quantified using green fluorescent signals from caspase-3/7-positive apoptotic cells normalized to cell density. Data represent the mean and SD of n = 3 biological replicates and are representative of at least two independent experiments.

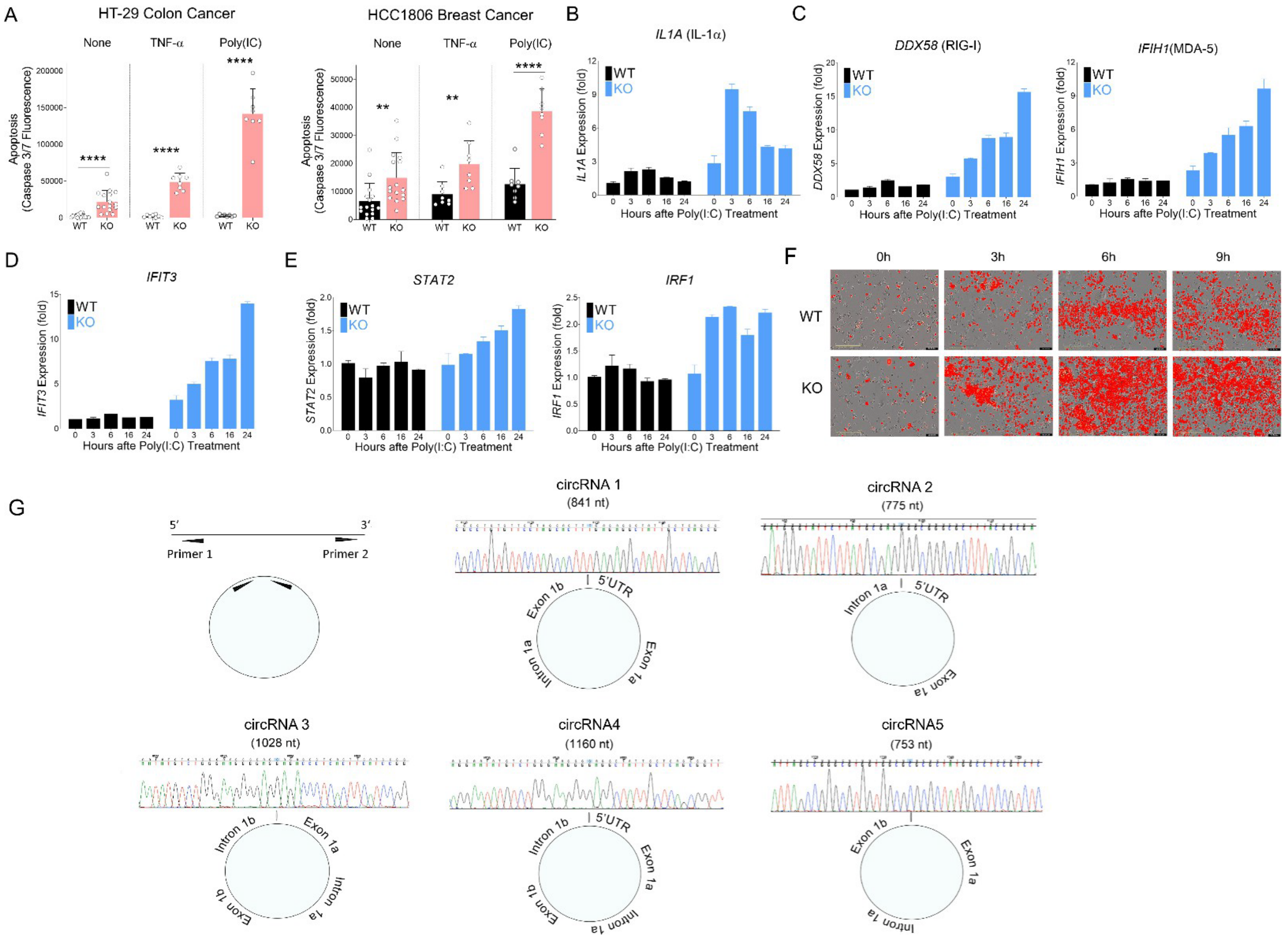

Supplementary Fig. S2. Increased Sensitivity to Poly (I:C) and Elevated Phagocytosis in *BRCA1P1*-Depleted Cells. **A**. Apoptosis of HT-29 and HCC1806 cells with *BRCA1P1*-wild type (WT) and knock-out (KO) genotypes was analyzed using the IncuCyte Live-Cell Imaging System. Apoptosis was quantified using green fluorescent signals from caspase-3/7–positive apoptotic cells normalized to cell density. Data represent the mean and SD of n = 8 biological replicates and are representative of at least two independent experiments. **B-E**. qRT-PCR analyses of antiviral gene expression 3-24 hours after 100 µg poly (I:C) treatment. Data represent the mean and SD of n=3-4 biological replicates. **F.** Phagocytosis cells were stained with pHrodo red, which is a fluorogenic dye that increases in fluorescence as the pH of its surroundings becomes more acidic. Representative images of pHrodo red-stained cells at 0, 3, 6, and 9 hours post co-culture. HT-29-WT and *BRCA1P1*-KO cells were co-cultured with M1-polarized THP-1 cells, and the number of Red fluorescent cells per well was measured. **G.** Two divergent primers were designed to amplify circular RNAs, as shown in the drawing. Sequencing of individual circular RNA clones showed at least 5 different formations of circular RNAs, ranging from 753 to 1160 nucleotides

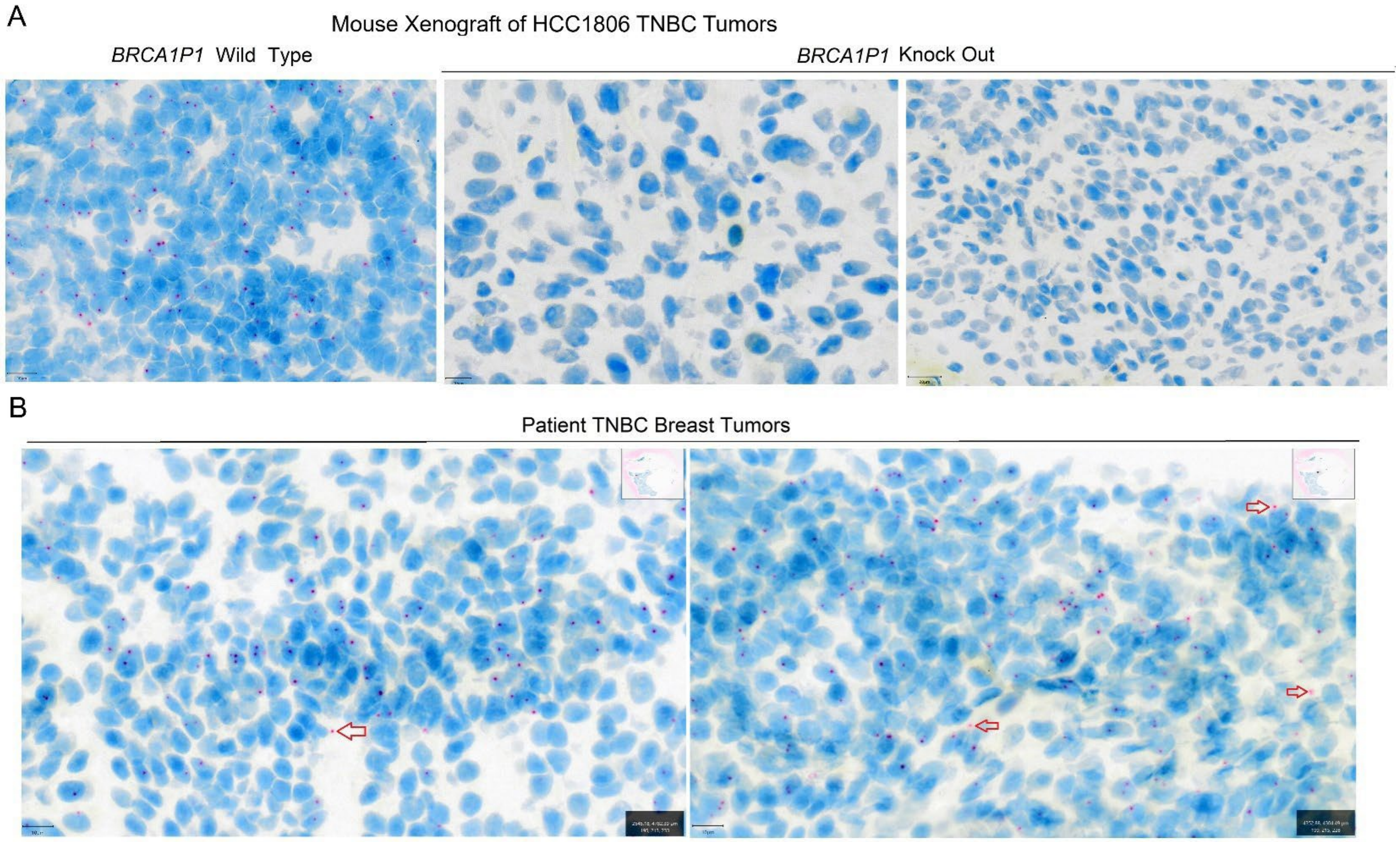

Supplementary Fig. S3. Detection of *BRCA1P1* RNA in Mouse Xenograft and Human TNBC Tumors Using BaseScope Technology. A. A single ZZ-pair probe specific for *BRCA1P1* was hybridized to mouse xenograft tumors derived from HCC1806 cells, yielding strong red signals in *BRCA1P1* wild-type tumors and no detectable signal in *BRCA1P1* knockout tumors; nuclei are counterstained with DAPI (blue). **B**. The vast majority of *BRCA1P1* signals were localized within the DAPI-stained nuclear area in patient TNBC tumors, with only a small fraction observed outside the DAPI signal, as indicated by red arrows.

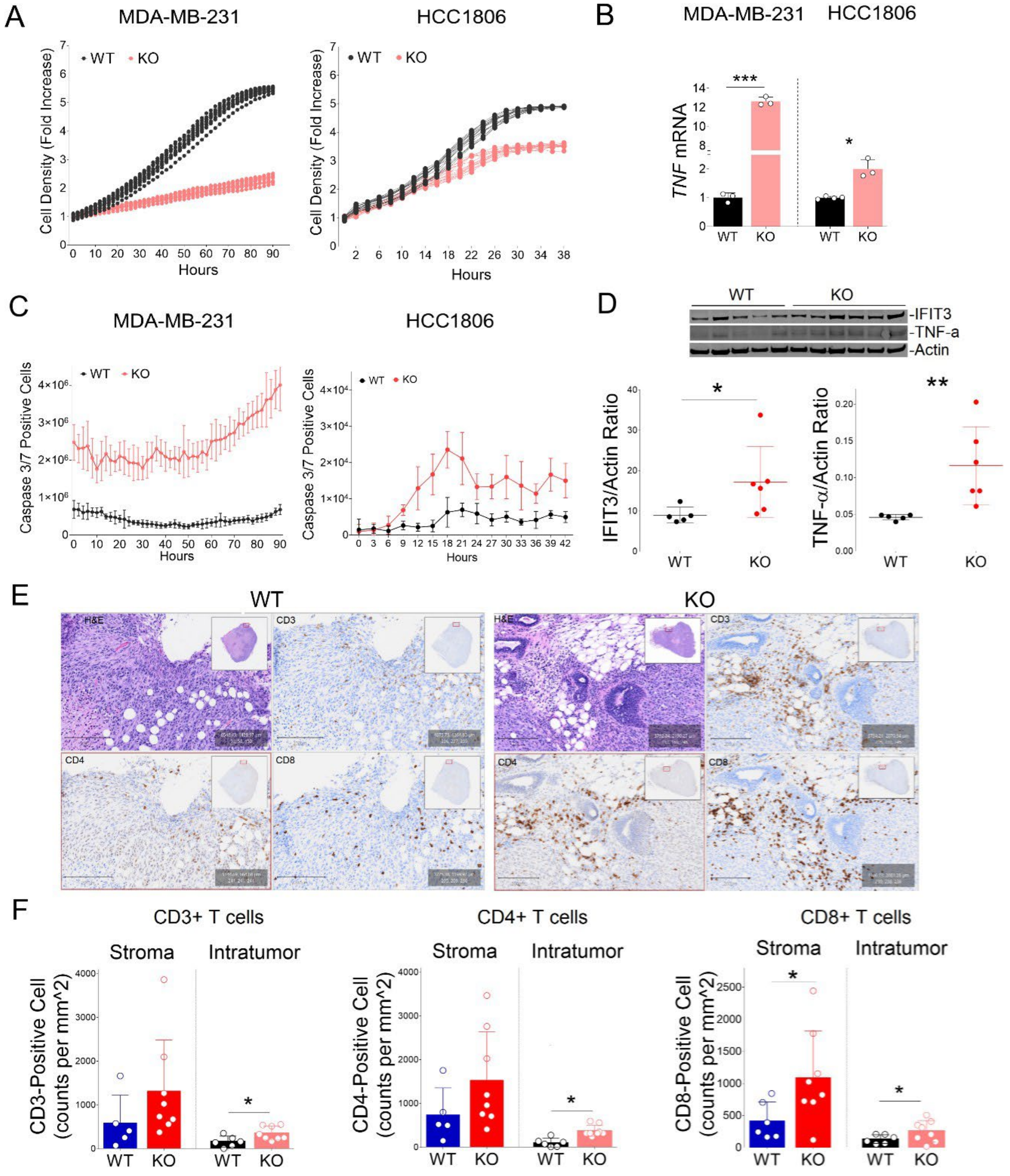

Supplementary Fig. S4. TNBC Cell Growth and Immune Infiltration in Humanized Mice A. Proliferation of MDA-MB-231 and HCC1806 TNBC cells with either *BRCA1P1* wild-type (WT) or knockout (KO) genotypes was analyzed using the IncuCyte Live-Cell Imaging System. Cell density values were derived from phase-contrast images of cells to evaluate cell proliferation. Fold increases in cell density were calculated compared to the cell density at 0 hour. Data represent n = 8-12 biological replicates and are representative of at least two independent experiments. **B**. qRT-PCR analyses of *TNF* mRNA expression in *BRCA1P1*-WT and KO cells. Data represent the mean and SD of n=3-4 biological replicates. **C**. Apoptosis of MDA-MB-231 and HCC1806 cells with *BRCA1P1*-WT and KO genotypes was analyzed using the IncuCyte Live-Cell Imaging System. Apoptosis was quantified using green fluorescent signals from caspase-3/7–positive apoptotic cells normalized to cell density. **D**. Western blot analysis of additional mice showed increased IFIT3 and TNF-α expression in *BRCA1P1*-KO tumors from HCC1806 xenograft mice. (*p<0.05; **p<0.01). **E**. Representative images of H&E (top left), and IHC of CD3- (top right), CD4- (bottom left) and CD8-positive (bottom right) cells infiltrated into *BRCA1P1*-WT and KO HCC1806 xenograft tumors. **F**. Quantification of T cell infiltration in both stroma and intratumoral regions of *BRCA1P1*-WT and KO HCC1806 xenograft tumors.

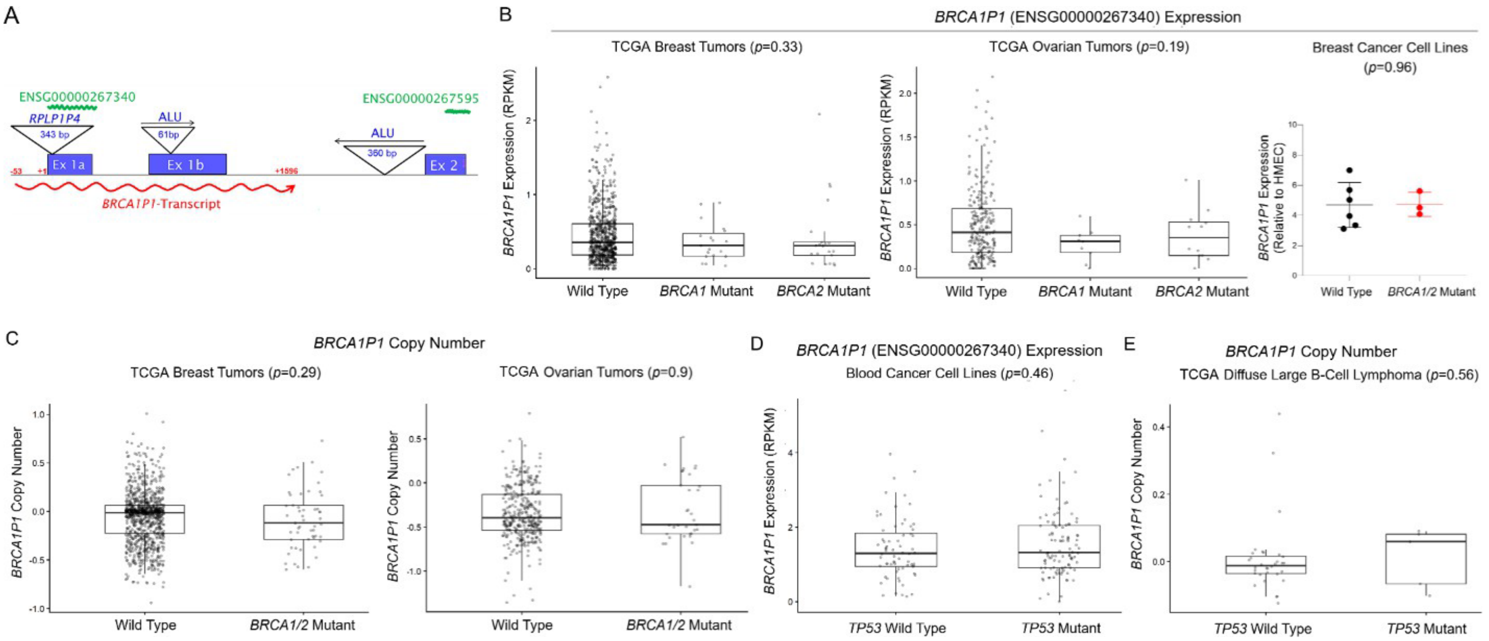

Supplementary Fig. S5. Public Data Analyses of *BRCA1P1* Expression and Copy Number by Mutational Status. RNA expression (RPKM or RSEM) and copy number (segment mean log2 ratio) were evaluated using TCGA PanCancer Atlas datasets for breast invasive carcinoma and ovarian serous cystadenocarcinoma. **A**. Schematic representation of the *BRCA1P1* gene structure and the positions of GENCODE/Ensembl-annotated transcripts. ENSG00000267340 was used as a proxy for *BRCA1P1* expression and compared between *BRCA1* wild-type and mutant tumors. **B**. *BRCA1P1* (ENSG00000267340) expression by *BRCA1* or *BRCA2* mutation status in breast tumors (*BRCA1/2* wild type, n = 752; *BRCA1* mutant, n = 19; *BRCA2* mutant, n = 21), ovarian tumors (*BRCA1/2* wild type, n = 248; *BRCA1* mutant, n = 9; *BRCA2* mutant, n = 12), and breast cancer cell lines (*BRCA1/2* wild type, n = 6; *BRCA1/2* mutant, n = 3). **C**. *BRCA1P1* copy number by *BRCA1/2* mutation status in breast tumors (*BRCA1/2* wild type, n = 941; *BRCA1/2* mutant, n = 53) and ovarian tumors (*BRCA1/2* wild type, n = 365; *BRCA1/2* mutant, n = 33). **D**. *BRCA1P1* expression by *TP53* mutation status in blood cancer cell lines (*TP53* wild type, n = 68; *TP53* mutant, n = 105). **E**. *BRCA1P1* copy number by *TP53* mutation status in diffuse large B-cell lymphoma (*TP53* wild type, n = 32; *TP53* mutant, n = 6). Expression and copy-number distributions are shown as boxplots with overlaid sample-level points; differences between mutant and wild-type groups were assessed using Kruskal–Wallis (**B**) or two-sided Wilcoxon rank-sum tests (**C-E**).

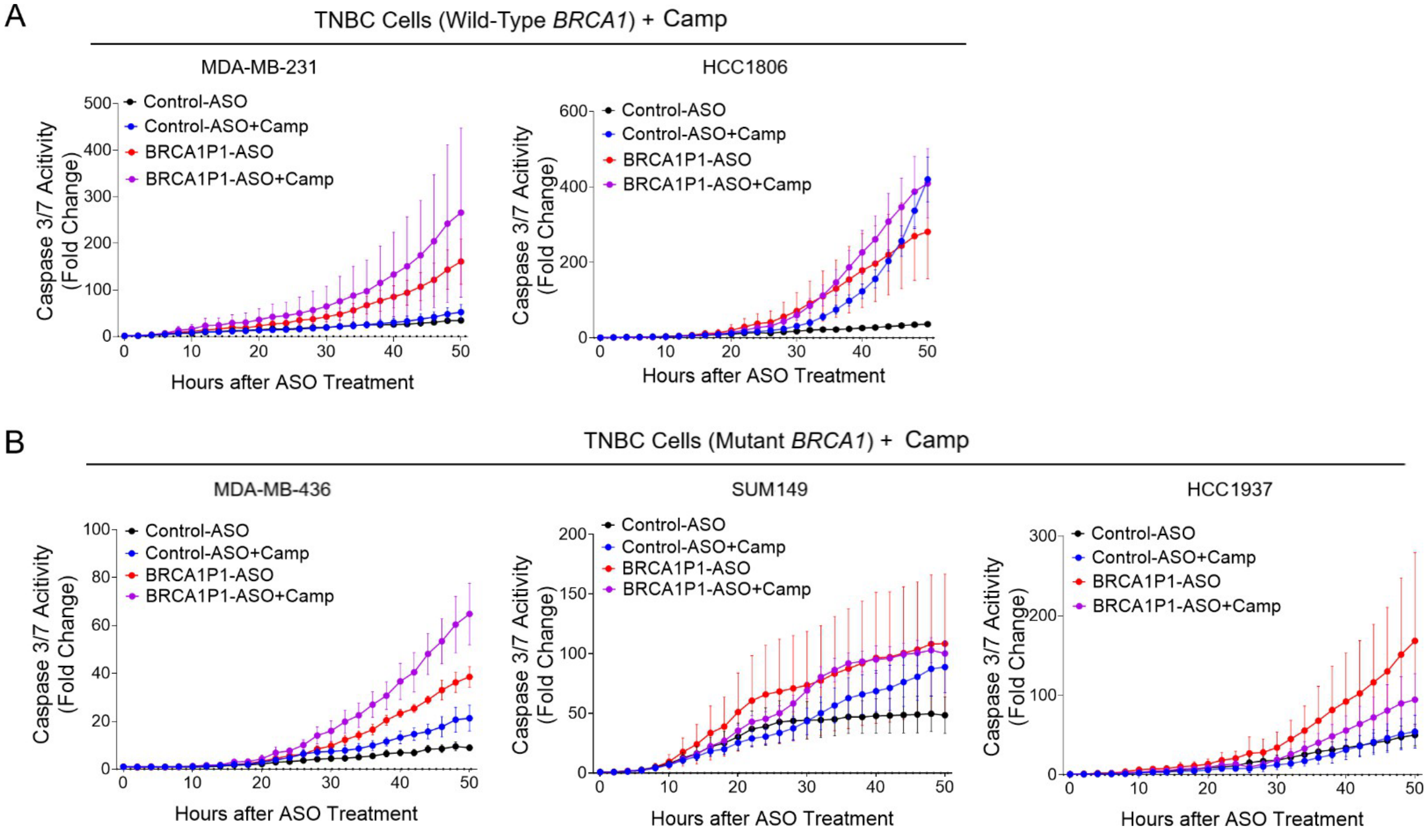

Supplementary Fig. S6. Camptothecin Sensitivity in *BRCA1*-mutated TNBC cells. A and B. Apoptosis was assessed in *BRCA1P1*-ASO and control-ASO treated TNBC cells harboring wild-type (**A**) or mutant *BRCA1* genotypes (**B**) following camptothecin (Camp) treatment (1 μM). Caspase-3/7-positive apoptotic cells (green fluorescence) were quantified and normalized to cell density. Data represent the mean and SD of n = 3 biological replicates and are representative of 2 independent experiments.

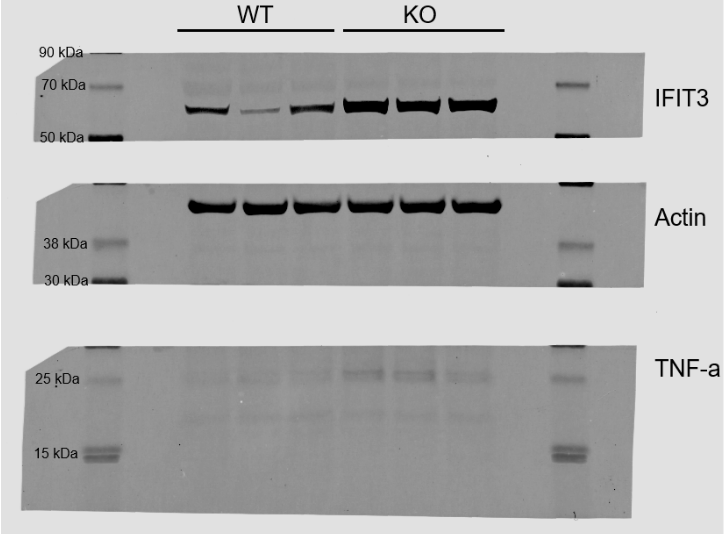

Supplementary Fig. S7. The Original Western Blot Image for Fig. 5C. Original Western blot image corresponding to Fig. 5C, showing target proteins (IFIT3 and TNF-α) and loading control (Actin) bands for the experimental and control conditions. The blot was acquired and scanned using Image Studio 6.1 (LI-COR).

References

1. Y. J. Han *et al.*, The BRCA1 Pseudogene Negatively Regulates Antitumor Responses through Inhibition of Innate Immune Defense Mechanisms. *Cancer Res* **81**, 1540-1551 (2021).
2. Y. J. Han *et al.*, LncRNA BLAT1 is Upregulated in Basal-like Breast Cancer through Epigenetic Modifications. *Scientific Reports* **8**, 15572 (2018).
3. Y. J. Han *et al.*, An enhancer variant associated with breast cancer susceptibility in Black women regulates TNFSF10 expression and antitumor immunity in triple-negative breast cancer. *Hum Mol Genet* **32**, 139-150 (2023).
4. Y. J. Han, S. F. Ma, M. S. Wade, C. Flores, J. G. Garcia, An intronic MYLK variant associated with inflammatory lung disease regulates promoter activity of the smooth muscle myosin light chain kinase isoform. *J Mol Med (Berl)* **90**, 299-308 (2012).
5. C. X. Liu *et al.*, Structure and Degradation of Circular RNAs Regulate PKR Activation in Innate Immunity. *Cell* **177**, 865-880 e821 (2019).
6. L. Han *et al.*, The Pan-Cancer analysis of pseudogene expression reveals biologically and clinically relevant tumour subtypes. *Nat Commun* **5**, 3963 (2014).

Table S1. Clinical Characteristics of Breast Cancer Patients Included in the BaseScope RNA-*ISH* Analysis

ID	Age	ER	PR	HER2	Gender	Subtype	Laterality	Neoadjuvant	Vital Status	Cancer Status	Clinical Stage Group	Pathologic Stage Group
R003	84	0	0	0	F	TNBC	1 Right - origin of primary	No Neoadjuvant	Alive	Remission	3A	3A
R021	34	0	0	1	F	HER2	2 Left - origin of primary	No Neoadjuvant	Alive	Remission	1	2B
R028	83	0	0	0	F	TNBC	2 Left - origin of primary	No Neoadjuvant	Deceased	Remission	2B	2B
R030	71	0	0	0	F	TNBC	1 Right - origin of primary	No Neoadjuvant	Deceased	Active Disease	1	2A
R034	80	1	1	0	F	ER	2 Left - origin of primary	No Neoadjuvant	Deceased	Remission	1	1
R035	58	1	1	0	F	ER	2 Left - origin of primary	No Neoadjuvant	Alive	Remission	2A	2A
R036	49	0	0	1	F	HER2	2 Left - origin of primary	No Neoadjuvant	Alive	Remission	3A	Unknown
R044	55	1	1	0	F	ER	2 Left - origin of primary	No Neoadjuvant	Alive	Remission	1	1

ER/PR/HER2 status: 0, negative; 1, positive

Table S2. Clinical Features of Patient-Derived Organoids from Breast Tumors

Sample ID	Race	Specimen Type	SUBTYPE	Tumor Grade	BRCA1	P53	Survival Status	Procedure Type	Organoid Notes
**S021**	White	Breast Tumor	TNBC	II	N/A	N/A	Deceased	Breast Biopsy	Robust Growth
**S035**	Black	Breast Tumor	TNBC	III	N/A	N/A	Alive	Mastectomy	Robust Growth
**S033**	White	Liver Metastasis	TNBC	Metastasis	N/A	N/A	Deceased	Liver Biopsy	Robust Growth
**S030**	Mixed	Pleural Effusion	ER+PR+HER2-	Metastasis	N/A	N/A	Deceased	Pleural Effusion	Robust Growth
